# Evaluation of Nanolipoprotein Particles (NLPs) as an In Vivo Delivery Platform

**DOI:** 10.1371/journal.pone.0093342

**Published:** 2014-03-27

**Authors:** Nicholas O. Fischer, Dina R. Weilhammer, Alexis Dunkle, Cynthia Thomas, Mona Hwang, Michele Corzett, Cheri Lychak, Wasima Mayer, Salustra Urbin, Nicole Collette, Jiun Chiun Chang, Gabriela G. Loots, Amy Rasley, Craig D. Blanchette

**Affiliations:** 1 Biosciences and Biotechnology Division, Lawrence Livermore National Laboratory, Livermore, California, United States of America; 2 School of Natural Sciences, University of California Merced, Merced, California, United States of America; Universidad de Castilla-La Mancha, Spain

## Abstract

Nanoparticles hold great promise for the delivery of therapeutics, yet limitations remain with regards to the use of these nanosystems for efficient long-lasting targeted delivery of therapeutics, including imparting functionality to the platform, *in vivo* stability, drug entrapment efficiency and toxicity. To begin to address these limitations, we evaluated the functionality, stability, cytotoxicity, toxicity, immunogenicity and *in vivo* biodistribution of nanolipoprotein particles (NLPs), which are mimetics of naturally occurring high-density lipoproteins (HDLs). We found that a wide range of molecules could be reliably conjugated to the NLP, including proteins, single-stranded DNA, and small molecules. The NLP was also found to be relatively stable in complex biological fluids and displayed no cytotoxicity *in vitro* at doses as high as 320 µg/ml. In addition, we observed that *in vivo* administration of the NLP daily for 14 consecutive days did not induce significant weight loss or result in lesions on excised organs. Furthermore, the NLPs did not display overt immunogenicity with respect to antibody generation. Finally, the biodistribution of the NLP *in vivo* was found to be highly dependent on the route of administration, where intranasal administration resulted in prolonged retention in the lung tissue. Although only a select number of NLP compositions were evaluated, the findings of this study suggest that the NLP platform holds promise for use as both a targeted and non-targeted *in vivo* delivery vehicle for a range of therapeutics.

## Introduction

The advent of nanotechnology has resulted in a variety of new possibilities for targeted delivery of therapeutic agents. In particular, delivery of therapeutic agents facilitated by nanoparticles is being implemented to solve several limitations of conventional drug delivery systems, including nonspecific bio-distribution and targeting, poor aqueous solubility, limited oral bioavailability, and low therapeutic indices [1]. Several types of nanoparticles have been developed to achieve targeted delivery of therapeutics, including inorganic nanoparticles [2], polymeric-based nanoparticles [3], polymeric micelles [4], dendrimers [5], liposomes [6], viral nanoparticles [7] and carbon nanotubes [8], each offering unique characteristics in nanoparticle composition, structure, and method of assembly. Despite the significant advantages these delivery vehicles provide over conventional drug delivery systems, there are still limitations with regards to the use of these nanosystems for efficient long-lasting targeted delivery of therapeutics, including *in vivo* stability, immunogenicity, targeting specificity, drug entrapment efficiency, long term storage, and toxicity [9]. One approach to address the issues associated with current nanoparticle platforms, particularly immunogenicity and toxicity, is to utilize a nanoconstruct that mimics supramolecular structures naturally present in the human body. One notable example of such a system is the lipoprotein class of nanoparticles, or high-density lipoproteins (HDLs), which are naturally present in most metazoan species and play an essential role in mammalian control of lipid metabolism [10]. These endogenous nanoparticles are utilized to transport hydrophobic cholesterol and triglycerides to cells through the circulatory system. The structure and function of HDLs *in vivo* have been studied for the past three decades, and methods for assembling several different compositionally distinct HDLs *ex vivo* [also called reconstituted HDLs (rHDLs), nanodiscs, or nanolipoprotein particles (NLPs)] have been developed [11]–[14]. The vast majority of the work on rHDLs and NLPs has been directed at both understanding the biology of such particles [15]–[18] as well as exploring their utility in solubilizing and stabilizing membrane proteins in discrete, native lipid environments [19]–[24]. However, the use of these particles for delivery of therapeutic drugs [25]–[28], diagnostic imaging [29], and vaccine and immunomodulation applications [30]–[33] has only recently been examined.

NLPs are nano-scale (8–25 nm) discoidal membrane bilayer mimetics that form through spontaneous self-assembly of purified lipoproteins and lipids [11], [12]. NLP formation and self-assembly is initiated by incubating detergent-solubilized lipids with apolipoproteins. Upon the removal of detergent, the lipid molecules assemble into nanoscale lipid bilayers that are stabilized at their periphery by lipoproteins. The amphipathic lipoproteins are oriented such that the lipophilic face interacts with the alkyl chains of the lipid bilayer, whereas the polar face is solvent-exposed. While the assembly of NLPs is facile, the diversity in both the lipid and protein [12], [24], [34] constituents illustrates the robust nature of the assembly process. In addition, due to the inherent amphipathic nature of lipid bilayers, the NLP platform is amenable to the incorporation of diverse lipids (in terms of both fatty acid chains and polar headgroups) and other hydrophobic or amphipathic molecules (e.g. cholesterol). The relative ease of forming NLPs through self-assembly, the ability to incorporate myriad lipophilic molecules within the NLP bilayer, and the diverse tool-kit of functionalized lipids either commercially available or readily synthesized suggest that NLPs are highly amenable to accommodate a disparate range of cargo molecules. Importantly, since these particles are naturally present in the human body, the NLP platform is less likely to result in issues facing other nanoparticle systems that are currently used for the targeted delivery of therapeutics, such as immunogenicity, stability in complex biological fluids, and toxicity.

Thus, to assess the potential of using NLPs as an *in vivo* platform for the delivery of therapeutics, we examined 1) the stability of the NLP in complex biological fluids, 2) the potential of conjugating multiple, different molecules of disparate physicochemical properties to the NLPs, 3) the *in vitro* cytotoxicity of the NLP platform in relevant cell types, 4) the *in vivo* acute toxicity of the NLP, 5) immunogenicity of the NLP and 6) the *in vivo* bio-distribution of the NLPs administered by five different routes. Although only a select number of NLP compositions (in terms of lipid constituents) were evaluated, the findings of this study suggest that the NLP platform holds promise for use as both a targeted and non-targeted *in vivo* delivery vehicle for a range of therapeutics.

## Materials and Methods

### Materials

1,2-dioleoyl-*sn*-glycero-3-phosphocholine (DOPC), 1,2-dimyristoyl-*sn*-glycero-3-phosphocholine (DMPC), 1,2-dioleoyl-*sn*-glycero-3-((N(5-amino-1-carboxypentyl)iminodiacetic acid) succinyl)(nickel salt) (NiLipid), and 1,2-distearoyl-*sn*-glycero-3-phosphoethanolamine-N-[folate(polyethylene glycol)-2000] (ammonium salt) (DSPE-PEG2000-folate) (PF) were purchased from Avanti Polar Lipids (Alabaster, AL). All other reagents were ordered from Sigma-Aldrich (St. Louis, MO). NHS-PEG4-DBCO was purchased from Click Chemistry Tools (Scottsdale, AZ). The cholesterol-modified oligodeoxynucleotide (cODN) (5′–TCAACATCAGTCTGATAAGCTA–tetraethyleneglycol–cholesterol–3′) was purchased from Integrated DNA Technologies (Coralville, IA). The C18-PEG6-N_3_ molecule was custom synthesized by Creative PEGworks (Winston Salem, NC). RPMI1640, Opti-MEM, fetal bovine serum, Alexa Fluor 647 NHS Ester (AF647), Alexa Fluor 750 NHS Ester (AF750), Alexa Fluor 488 5-Carboxamido-(6-Azidohexanyl), Bis(Triethylammonium Salt) (AF488-azide), primary antibodies and secondary antibodies were obtained from Invitrogen (Carlsbad, CA). ELISA kits for cytokine analysis were purchased from R&D systems (Minneapolis, MN). The kits for LDH and MTT analysis were purchased from Promega (Madison, WI) and Invitrogen.

### Protein expression and purification

The expression clone for the 22 kDa N-terminal fragment of human apolipoprotein E4 (apoE422k, kindly provided by Dr. Karl Weisgraber) featuring a cleavable His-tag [35] was expressed and purified as previously described [11], [36]. The expression clone for the *Y. pestis* protein used in this study, LcrV, was expressed and purified as previously described [30].

For expression of FTN_0841 (0841), the full length FTN_0841 ORF was amplified from genomic DNA isolated from *F. tularensis* subsp. *novicida* strain U112 using gene-specific primers (forward primer: 5′ - ctcgaattcCATATGAAAAATGTCTTAATGGTTACC, reverse primer 5′ - ggaattGGATCCATTAAATAGTGATTGTTTTATTGCTT) containing engineered 5′ NdeI and 3′ BamHI endonuclease sites. The PCR-amplified 0841 gene was cloned into a modified pETBlue expression plasmid, pETBlueER by directed cloning. The resulting plasmid encoded a native 0841 protein appended with a C-terminal His-tag (GSLEHHHHHH). Expression plasmids encoding 0841 were propagated in *E. coli* DH5α-Ti cells, and colonies carrying the plasmid were selected on LB/agar plates with 100 µg/ml ampicillin. Plasmid DNA was then transformed into BL21(DE3)pLacI competent cells (Novagen) for expression. Individual colonies were grown in LB media containing 100 µg/ml ampicillin at 37° to O.D. 600 of ∼0.6 and protein expression was induced by the addition of isopropyl thiogalactoside (IPTG) at a final concentration of 1 mM for 3 hrs. The bacteria were pelleted by centrifugation at 4000× g and frozen. The thawed bacterial pellet was resuspended in 50 mM sodium phosphate, 300 mM sodium chloride, 10 mM imidazole, pH 8.0 and lysed for 20 min in a high-pressure homogenizer (Emulsiflex-C5, Avestin). Lysates were centrifuged at 8,000× g to remove insoluble material. 0841 was initially purified from the clarified supernatant by nickel affinity chromatography using a 5 ml HisTrap FF Column (GE Healthcare) on an AKTA FPLC (GE Healthcare). The column was washed with 50 mM NaH_2_PO_4_, 300 mM NaCl, 20 mM imidazole pH 8, and the His tagged protein was eluted with 50 mM NaH_2_PO_4_, 300 mM NaCl and 250 mM imidazole pH 8.0. The eluted protein was further purified and buffer exchanged into 10 mM Hepes, 50 mM NaCl pH 7.5 on a Superdex 75 HiLoad 26/60 column (GE Healthcare). Fractions containing protein were analyzed by SDS-PAGE stained with SYPRO Ruby (Invitrogen) to assess purity and concentrated using Vivaspin 20 5 kDa molecular weight cut-off MWCO (Sartorius) to a final concentration of 11 mg/ml.

### NLP assemblies

NLPs were assembled according to a previously reported procedure [11], [36] with slight modifications. For all NLP assemblies, the total lipid-to-apoE422k molar ratio was 80∶1. This ratio was selected since particles assembled under these conditions consistently have an average diameter ranging from 20–25 nm, as assessed by size exclusion chromatography (SEC) and atomic force microscopy (AFM) [14], [34], [37], [38]. Briefly, lipids were either prepared or obtained in chloroform and aliquoted into glass vials. Chloroform was then removed using a stream of N_2_ under agitation to form a thin lipid film. Lipids were solubilized in PBS buffer (137 mM sodium chloride, 2.7 mM potassium chloride, 10 mM phosphate buffer, pH 7.4) using 30 mM sodium cholate. After addition of the apoE422k (150 µM in final assembly volume), samples were incubated at 23.8°C for at least 2 hours. Assemblies were dialyzed overnight against PBS to remove cholate. Samples were subsequently analyzed and purified by SEC (Superdex 200, 10/300 GL column, GE Healthcare, Piscataway, NJ) in PBS buffer (0.5 mL/min flow rate). The exclusion limit of the column was determined with Blue Dextran 2000. SEC fractions (500 µl) were collected every 60 s. SEC fractions containing homogeneous NLP populations were concentrated using 50 kDa MWCO spin concentrators (Sartorius). The apoE422k concentration was determined using the Advanced Protein Assay Reagent (Cytoskeleton Inc., Denver, CO), where BSA was used as the standard. The concentrated NLP samples were then stored at 4°C until further use.

### Labeling the NLPs with Alexa Fluor dyes

NLPs were labeled with either AF647 (stability experiments) or AF750 (biodistribution experiments) by incubating the NLPs with the respective reactive dye for at least 2 hrs (5∶1 dye∶NLP molar ratio). The reaction was performed in PBS buffer containing 5 mM sodium bicarbonate, pH 8.2. After completion of the reaction, 10 mM Tris pH 8.0 was added to quench any unreacted dye and incubated for 30 minutes. The samples were then run on SEC (Superdex 200 PC 3.2/30 column, GE Healthcare, Piscataway, NJ) to purify out the labeled NLP from unreacted dye (0.15 mL/min flow rate). The SEC fractions corresponding to the NLP were then pooled and concentrated using 50 kDa MWCO spin concentrators. The apoE422k concentration was determined using the Advanced Protein Assay Reagent (Cytoskeleton Inc., Denver, CO), where BSA was used as the standard. The concentrated NLP samples were then stored at 4°C until further use.

### SEC analysis of NLP stability in complex biological fluids

AF647-labeled NLPs were purified to homogeneity with diameters averaging 23.5 nm [37]. NLP samples were incubated with increasing serum concentrations and subsequently analyzed by SEC (Superdex 200 PC 3.2/30 column, GE Healthcare) in PBS buffer. A flow rate of 0.15 ml/min was used to ensure no overlap in the elution of disassembled apoE422k and intact NLP. The NLPs labeled with AF647 were monitored at an absorbance wavelength of 600 nm to avoid interfering absorbance at 280 nm from serum proteins and constituents. NLP peak integration was used to assess NLP disassembly as a function of incubation time using instrument software (LC Solutions, Shimadzu). These experiments were performed with NLPs made with either 100% DOPC or 100% DMPC.

### Click chemistry

The recombinant protein 0841 was covalently attached to N_3_NLPs through a click reaction that involved functionalizing the 0841 protein with a NHS-activated strained alkyne molecule dibenzylcyclooctyne (DBCO). 0841 was functionalized with DBCO by incubating the protein with DBCO for at least 2 hrs (5∶1 DBCO:0841 molar ratio) in PBS buffer containing 5 mM sodium bicarbonate, pH 8.2. After the overnight incubation, 10 mM Tris pH 8.0 was added and left to incubate for 30 minutes to quench any unreacted DBCO. The 0841 DBCO-functionalized protein (0841-DBCO) was then purified from unreacted DBCO by SEC (Superdex 200 PC 3.2/30 column (GE Healthcare, Piscataway, NJ). The SEC fractions corresponding to the 0841-DBCO peak were pooled and concentrated as described above. The 0841-DBCO concentration was then measured using a NanoDrop ND-1000 spectrophotometer (ThermoScientific, Lafayette, CO) at an absorbance of 280 nm (theoretical extinction coefficient – 28420). Successful covalent attachment of the DBCO to the 0841 protein was assessed based on conjugation between 0841-DBCO and an azide-functionalized fluorophore (AF488-azide). AF488-azide was incubated with 0841 and 0841-DBCO overnight in PBS buffer at a AF488-azide:protein molar ratio of 5. After overnight incubation, the samples were analyzed by SEC, measuring absorbance at both 280 nm and 495 nm. To evaluate covalent attachment of 0841-DBCO to N_3_NLPs, 0841-DBCO was incubated with N_3_NLPs overnight in PBS buffer at various 0841-DBCO:N_3_NLP ratios. After overnight incubation, conjugation was evaluated by SEC as described below. For the controls, 0841-DBCO was incubated with NLPs lacking an azide functional group and 0841 was incubated with N_3_NLPs under the same conditions described above.

### Analysis of conjugation of biological molecules to the NLP

Due to the significant size difference between the NLPs and free protein, SEC was used as a quantitative tool to assess conjugation of biological molecules to the NLP. The various NLP compositions used in these studies are described in the Results and Discussion section. NLP samples were analyzed by SEC (Superdex 200 PC 3.2/30 column, GE Healthcare) in PBS buffer. A flow rate of 0.15 ml/min was used to ensure no overlap in the elution of unbound protein and NLP. The samples were monitored and detected at an absorbance wavelength of 280 nm. For the protein conjugation experiments, purified NLP fractions were analyzed by SDS-PAGE, using SYPRO Ruby protein gel stain for visualization. Densitometry was used to quantify conjugated protein, using appropriate 0841 and apoE422k protein standards. Previously, computational modeling of apoE422k containing NLPs indicated that NLPs that were 23.5 nm in diameter have 6 apoE422k per NLP. Therefore, in these experiments, the NLP concentration was calculated based on the apoE422k concentration by assuming that each NLP contained 6 apoE422k scaffold proteins [11], [37].

### Quantification of the incorporation of cholesterol-modified oligodeoxynucleotide (cODN) and DPSE-PEG2000-folate (PF) into NLPs

NLPs were assembled in the presence of various concentrations of cODN and PF and purified by SEC. The amount of cODN incorporation was determined by first quantifying the apoE422k concentrations using the Advanced Protein Assay Reagent (Cytoskeleton Inc., Denver, CO), with BSA as the standard. NLPs without the cODN were then prepared and diluted to the same apoE422k concentration as the cODN∶NLPs. The absorbance at 260 nm of both the cODN∶NLP and NLP samples (at identical apoE422k concentrations) were measured using a NanoDrop ND-1000 spectrophotometer (ThermoScientific, Lafayette, CO). The contribution of absorbance at 260 nm from the cODN in the cODN∶NLP samples was then calculated by subtracting the total absorbance of the NLP alone sample from the cODN∶NLP at 260 nm. The concentration of the cODN was then calculated from this normalized absorbance value using the extinction coefficient of the cODN (ε_260_ = 220,000 M^−1^·cm^−1^). Folate has a characteristic intrinsic absorption peak at 368 nm, which was used to quantify the amount of PF incorporated into the PF∶NLPs [39]. A standard curve of PF was generated by measuring the absorption of PF samples at 368 nm over a concentration range of 0.05–1 mg/ml, which resulted in a near linear relationship. The amount of PF incorporated into the NLP was then calculated by measuring the absorption of the PF∶NLP samples at 368 nm and fitting that value to the generated standard curve.

### Isolation of primary splenic immune cells

Spleens were harvested from male BALB/c mice (4–6 weeks old), and single cell suspensions of splenoctyes were generated as follows. Briefly, spleens were injected with 200–500 µl of a solution of liberase (1.67 U/ml, Roche) and DNAse I (0.2 mg/ml, Roche) in RPMI +10% FBS and incubated for 30 minutes at 37°C before manual dissociation through 70 µm filters. Red blood cells were lysed by incubation in 1 ml ACK lysis buffer (Life Technologies) for 5 minutes at room temperature. The single-cell suspension of splenocytes was then sorted by positive selection into CD11c+ (dendritic cell (DC)), CD5^+^ (T cells), CD19^+^ (B cells), CD49b^+^ (natural killer cells (NK)) and CD11b^+^ (macrophages) populations using MACS bead sorting according to the manufacturer's instructions (Miltenyi Biotech). All experiments were conducted after review and approval by the Institutional Animal Care and Use Committee at Lawrence Livermore National Laboratory.

### Cytotoxicity measurements

The cytotoxicity of the NiNLP platform (35% NiLipid and 65% DOPC) in J774A.1 cells (J774, mouse macrophage cell line), primary mouse splenocytes (macrophages, dendritic cells (pDCs), natural killer (pNK) cells, B cells (pBC), T cells (pTC)) and HEP-G2 (human hepatic carcinoma) cells were measured using a LDH-based viability assay, whereas the cytotoxicity of the NLP against UMR (mouse osteoblastic cell line) cells was measured using the MTT-based viability assay. For the LDH-based assay, 0.5×10^6^ cells were plated into 24 well plates in 0.5 ml of Opti-MEM media (Invitrogen). After incubation with varying concentrations of NiNLPs for 24 hours, LDH levels were measured using the CytoTox96 non-radioactive cytotoxicity assay from Promega (Madison, WI) following the manufacturer's instructions. Briefly, 50 µl of the cell supernatant was added to 50 µl of LDH substrate and incubated for 10 minutes. After this incubation period, 50 µl of the stop solution was added, and the sample absorbance was measured at a wavelength of 490 nm. The data was normalized to cells that were just treated with PBS (set at 100% cell viability). Cells subjected to multiple freeze-thaw cycles were used as positive controls of the assay. For the UMR cytotoxicity experiment (MTT-based assay), 1×10^4^ cells were seeded into 96-well plates in 200 µl Opti-MEM media. The cells were then incubated with different NLP concentrations for 24 hrs. The media was replaced with 150 µl of fully supplemented F-12 MEM and incubated for 4 hr. The wells were then washed with fresh F-12 MEM media and 10 µl of a 12 mM stock solution of MTT was added. The plates were incubated at 37°C for 4 hours. 100 µl of an SDS-HCl solution (0.1 M SDS) was added to each well and mixed thoroughly with a pipette. The plates were then incubated at 37°C overnight in a humidified incubator and the absorbance at 570 nm of each well was measured. All cytotoxicity experiments were performed with NLPs consisting of 35% Ni-Lipid and 65% DOPC.

### In vivo acute toxicity and histological analysis

To evaluate the acute toxicity of the NiNLP (35% Ni-Lipid and 65% DOPC), groups of 6 five-week-old BALB/c mice (3 males and 3 females) were administered the NiNLP via the intraperitoneal (i.p.) or intranasal (i.n.) route daily for 14 days. The daily NiNLP dose was 25 µg (based on apoE422k protein), and inocula for i.p. and i.n. administration were delivered in a total volume of 100 µl and 30 µl, respectively, in PBS. This dose was selected based on previous studies where the NiNLP platform was used as an antigen delivery vehicle for vaccine applications [30], [32]. The weights of the mice were recorded daily each morning. Responsiveness and activity level were the two behavioral criteria used to assess overall animal health during the course of repeated treatment. The responsiveness of the mice was evaluated by tapping the side of the cage and observing movement, whereas activity level was assessed based on their attempts to evade handling prior to weighing. After the 14-day period, the mice were euthanized, and liver, kidney, lung and spleen were harvested. The weight of each organ was recorded and normalized to the weight of the mouse, which was recorded just prior to euthanasia. Mouse tissues were excised, fixed in 10% neutral buffered formalin (NBF) for 72 hours at 4°C, then dehydrated and processed for paraffin embedding. Tissues were embedded in paraffin wax, cut to 6 µm thickness, and stained with hematoxylin and eosin (H&E) for imaging. Slides were mounted with Permount, coverslipped, and imaged using a QImaging QIClick camera with a Leica DM5000B compound microscope. Images were acquired and adjusted using Image Pro Plus V7.0 software. All experiments were conducted after review and approval by the Institutional Animal Care and Use Committee at Lawrence Livermore National Laboratory.

### Immunogenicity

To determine the immunogenicity of the NiNLP (35% Ni-Lipid and 65% DOPC), groups of 10 BALB/c mice (5 males and 5 females) were inoculated with the NiNLP (25 µg) via the i.p. and i.n. routes. As a positive control, animals were inoculated with a formulation of LcrV (10 µg)+CpG (5 µg). Inocula for i.p. and i.n. administration were delivered in a total volume of 100 µl and 30 µl, respectively, in PBS. Serum was collected 3 weeks post-immunization via the saphenous vein. The collected sera were pooled from each animal in a given experimental group and analyzed by ELISA as described previously [30]. Immulon 2 HB microtiter plates (Thermo Labsystems, Franklin, MA) were coated with the appropriate antigen (LcrV or apoE422k; 200 ng/well) then incubated with sera at increasing dilutions for 1 hour. Goat anti-mouse IgG HRP-conjugated antibody (KPL Inc., Gaithersburg, MD) was added to the plates for 1 hour, and the bound HRP was detected by incubation with TMB (Sigma), prior to quenching after 5 min with 1 M HCl. The reaction product was measured spectrophotometrically at 450 nm, and values were corrected for background activity detected from wells that received diluent in place of sera. The titration curves were then fit to a power function in MS Office Excel 2010, and titers were calculated from the fit function using a cutoff absorbance value of the average background O.D.+3 S.D. All experiments were conducted after review and approval by the Institutional Animal Care and Use Committee at Lawrence Livermore National Laboratory.

### Biodistribution

NiNLPs (35% Ni-Lipid and 65% DOPC) were labeled with AF750 as described above. Groups of three mice were then immunized with 50 µg of NLP via i.p., i.n., intravenous (i.v.) intramuscular (i.m.), and subcutaneous (s.c.) administration. After predetermined time points, the mice were euthanized, and spleen, kidney, liver and lungs were removed. The fluorescence intensities in the excised organs were analyzed using a Kodak image station 4000R digital imaging system (Rochester, NY) at excitation and emission wavelengths of 750+/−20 nm and 790+/−20 nm, respectively. The fluorescence values of each organ were then normalized to the organ weight after subtracting for background fluorescence. The background fluorescence of each organ was determined from organs that had been harvested from control mice inoculated with PBS. All experiments involving animals were conducted after review and approval by the Institutional Animal Care and Use Committee at Lawrence Livermore National Laboratory.

## Results and Discussion

### NLP stability in complex biological fluids

**Figure 1:**
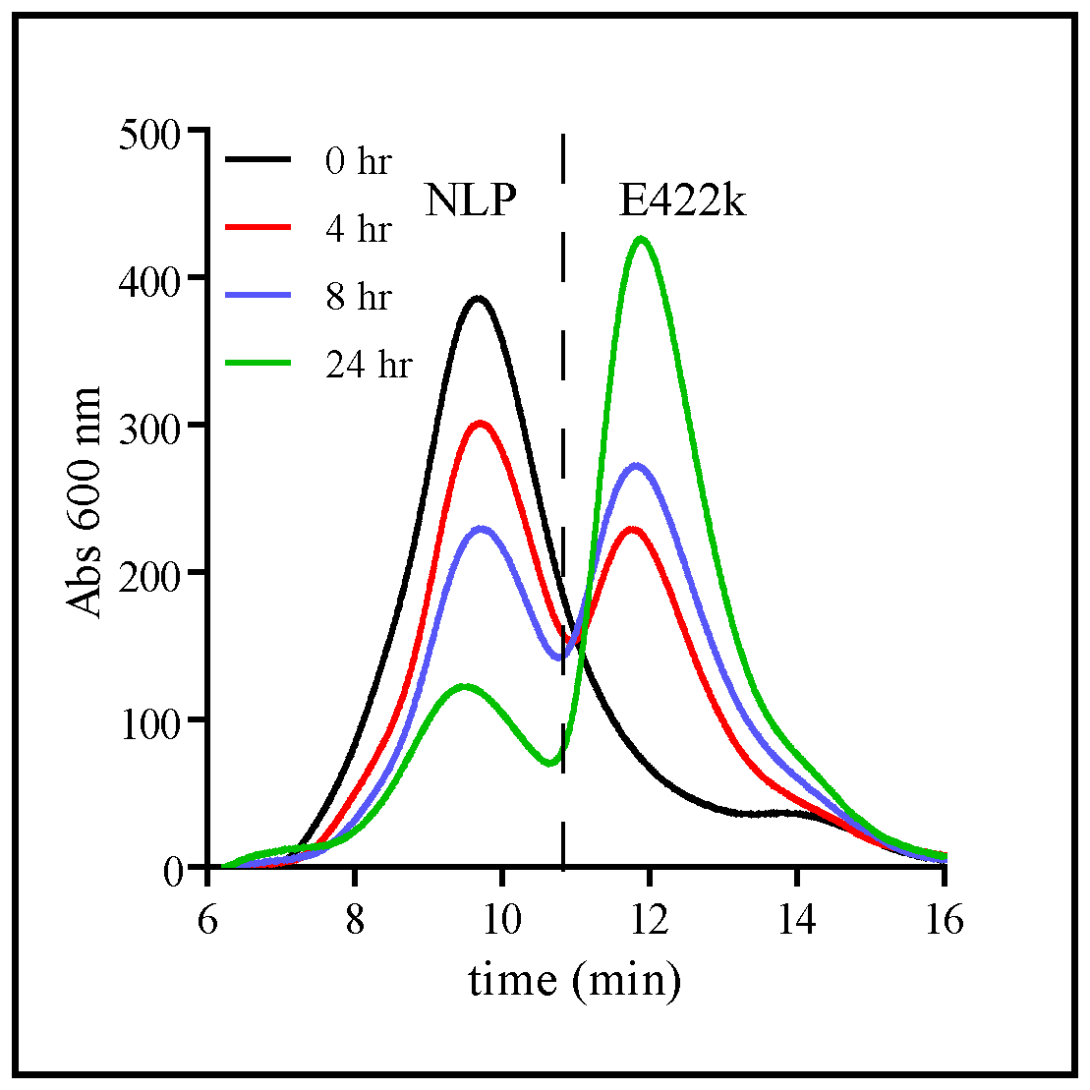
Stability of DMPC∶NLPs in 20% serum assessed by SEC over 24 hrs at 25°C. The indicated peaks correspond to intact NLP (t_R_ 9.5 min) and free apoE422k scaffold protein (t_R_ 12.2 min). Absorbance of AF647-labeled apoE422k absorbance was monitored at 600 nm.

**Figure 2:**
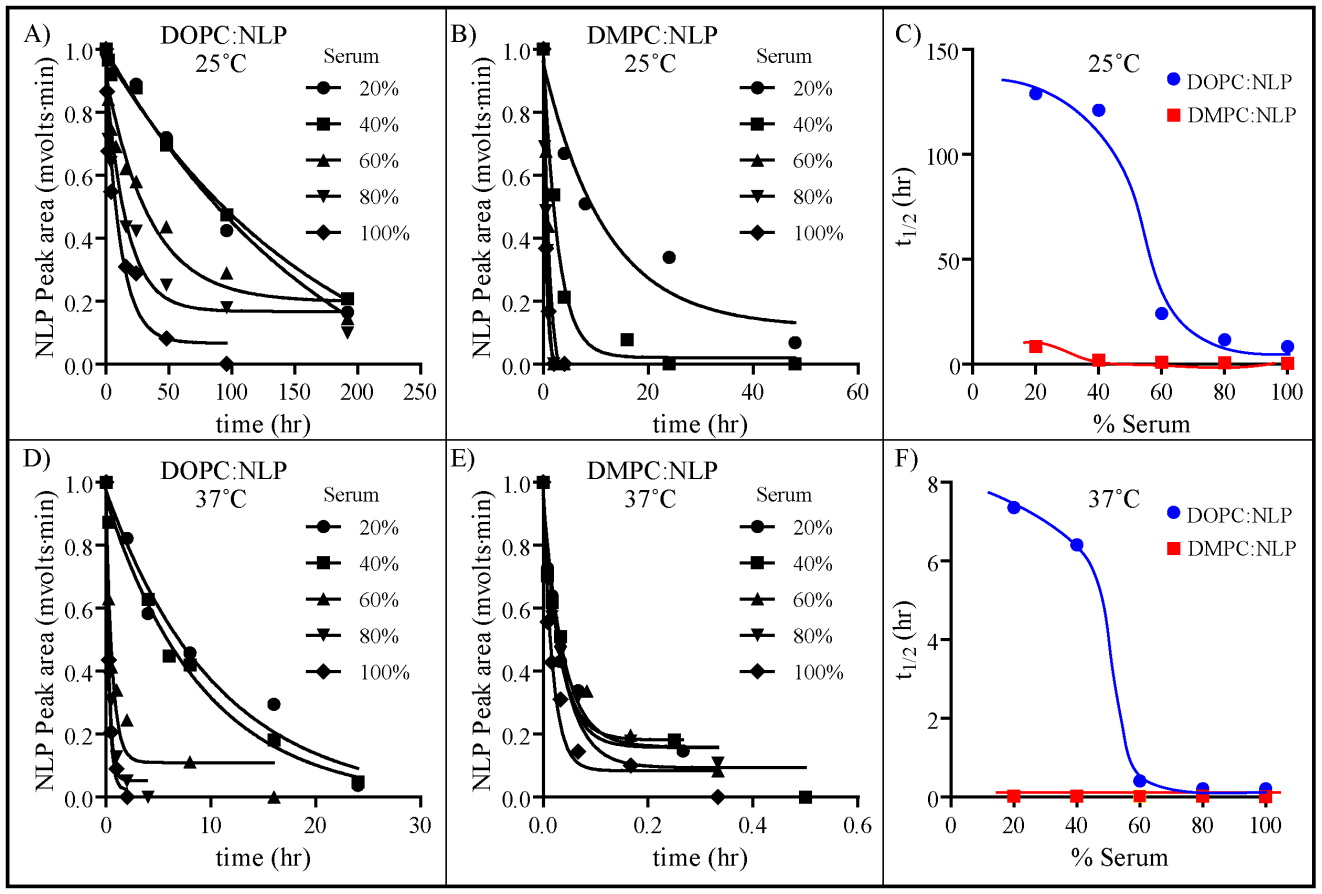
Stability of NLPs as a function of lipid content, temperature, time, and serum concentration. Integrated NLP peak area of the SEC chromatograms for A) DOPC∶NLPs incubated at 25°C and B) DMPC∶NLPs incubated at 25°C. C) t_1/2_ of the DOPC∶NLPs (blue line) and DMPC∶NLPs (red line) incubated at 25°C. Integrated NLP peak area of the SEC chromatograms for D) DOPC∶NLPs incubated at 37°C and E) DMPC∶NLPs incubated at 37°C. F) t_1/2_ of the DOPC∶NLPs (blue line) and DMPC∶NLPs (red line) incubated at 37°C. AF647-labeled apoE422k absorbance was monitored at 600 nm.

A key attribute necessary for any nanoparticle-based delivery vehicle is stability in complex biological fluids. Some of the drawbacks associated with current nanoparticle technologies include lack of stability in complex biological fluids [40], [41] or loss of function do to non-specific adsorption of serum proteins onto the particle surface (e.g. biomolecule corona) [42], [43]. Therefore, SEC was used to evaluate the stability and non-specific protein coating of the NLP platform in serum concentrations ranging from 20 to 100% in media (RPMI 1640) (spanning relevant *in vitro* and *in vivo* conditions) (Figure 1). To facilitate monitoring of NLP size and integrity by SEC, NLPs were labeled with AF647 to provide a spectrophotometric signature unperturbed by the intrinsic absorbance of serum proteins and constituents. The retention time (t_R_) of the intact NLP was distinct from free apoE422k released from dissociated NLPs (9 min vs. 12 min, respectively), and thus the stability and integrity of the NLP could be readily followed over time by SEC. Two different types of NLPs were assembled to interrogate the effect of NLP lipid composition on NLP stability: one consisting of 100% DMPC (fully saturated lipid – DMPC∶NLP) and the other 100% DOPC (mono-unsaturated lipid – DOPC∶NLP). These lipid compositions were chosen for their disparate bilayer properties and their preferential use in NLP-based applications. We also examined the effect of temperature on NLP stability (25°C vs. 37°C). Figure 1 shows the SEC chromatograms of DMPC∶NLPs incubated in 20% sera for 0, 4, 8 and 24 hr at 25°C. At 0 hr, only a single peak, corresponding to intact NLP (t_R_ ∼7.8 min) was observed. At longer incubation times, dissociated apoE422k (t_R_ 12 min) was observed concomitant with a decrease in the intensity of the intact NLP peak, indicating a gradual dissociation of the NLP. These chromatograms were used to quantify the rate of NLP dissociation by integrating the NLP peak area. Figure 2A and 2B show the dissociation of the DOPC∶NLPs and DMPC∶NLPs, respectively, at 25°C as a function of the different sera concentrations. As can be observed from these traces, the DMPC∶NLPs were considerably less stable as a function of time than the DOPC∶NLPs. To quantify this difference, the integrated areas of the NLP and apoE422k peaks in Figure 2A and 2B were fit to an exponential decay function and the half-life of the particle (t_1/2_) under each condition was calculated (Figure 2C). Based on these analyses, we discerned a measurable decrease in the NLP stability at increasing sera concentrations, whereby the DMPC∶NLPs were significantly less stable than the DOPC∶NLPs at all sera concentrations tested. At 25°C, the maximum t_1/2_ of the DOPC∶NLPs was 128.8 hrs (20% sera), whereas the maximum t_1/2_ of the DMPC∶NLPs was 8.3 hrs (20% sera) To examine the stability of these particles under more biologically relevant conditions, these experiments were repeated at 37°C (Figure 2D–2F). Overall, the DMPC∶NLPs were considerably less stable than the DOPC∶NLPs at all sera concentrations tested (Figure 2F). One possible reason for the difference in NLP stability between these two particles may be due to lipid-lipid and lipid-scaffold interactions. Although the structure of the NLP has long been reported to be a static discoidal structure, more recent studies have indicated that the NLP structure is more dynamic in solution and may adopt multiple different conformations ranging from discoidal to spherical [44]. DOPC lipid bilayers are more loosely packed than DMPC bilayers due to the presence of the unsaturated double bond in the acyl chain of the lipid. Thus, a loosely packed structure may facilitate flexibility in the three dimensional morphology of the NLP, i.e. allowing it to more readily adopt these different conformations relative to the more packed structure of DMPC-based NLPs, which may explain this increase in stability of the DOPC-based NLPs. However, further studies would be needed to conclusively determine the reasons for this increased stability.

It is also worth noting that regardless of the serum concentration, no significant shift in the NLP t_R_ was observed, suggesting that an increase in particle size due to non-specific serum protein adsorption or protein corona formation did not occur. While the peak t_R_ did slightly decrease with time under each serum condition (∼7.8 min to ∼6.8 min), this decrease was relatively small and was likely caused by a rearrangement in NLP size due to the loss of total apoE422k associated with the particles during degradation, as has been documented with similar NLP constructs [14]. These data suggest that NLPs do not have significant non-specific interactions with serum protein, a challenge faced by many other nanoparticle platforms, including inorganic nanoparticles [21].

Based on the superior stability of DOPC∶NLPs in these complex biological fluids, DOPC was utilized as the primary lipid constituent (referred to as the helper lipid) in all subsequent experiments.

### Multifunctional NLPs

**Figure 3:**
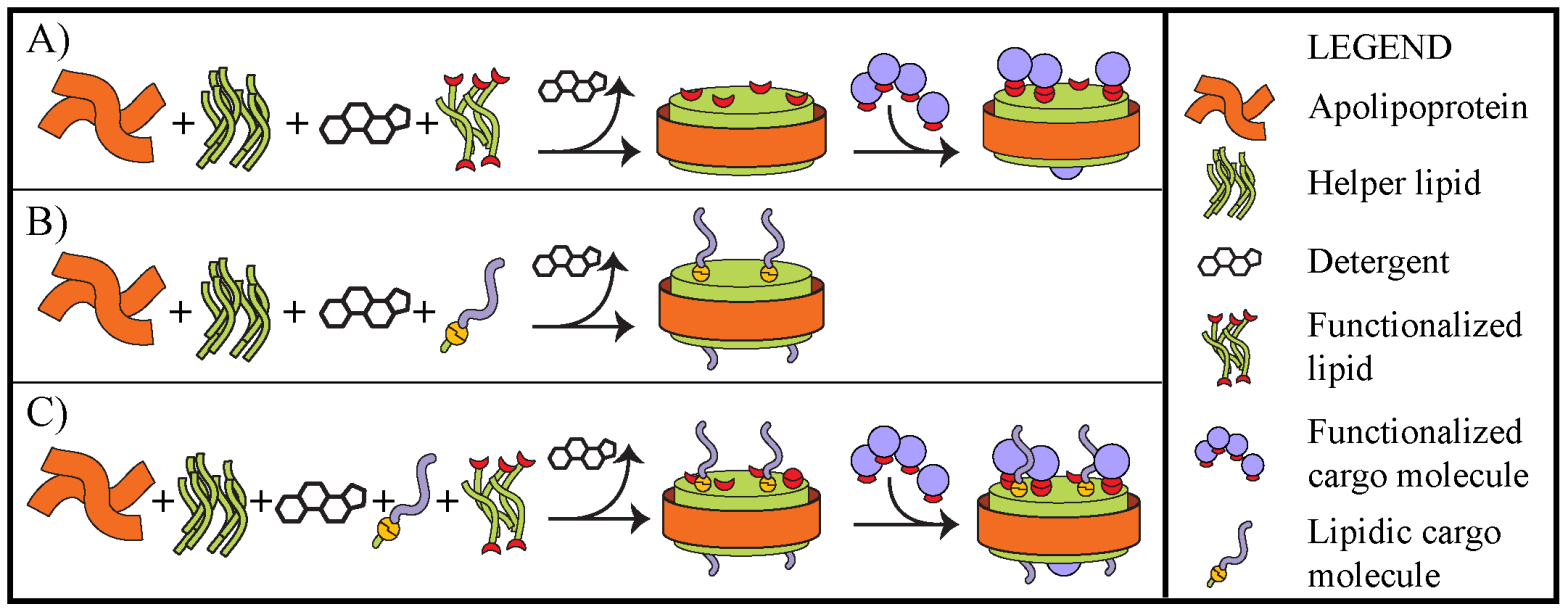
Illustration of strategies for conjugating biological or therapeutic cargo molecules to the NLP platform. Conjugation to NLPs can be achieved using A) lipids bearing functional head groups (e.g. chelated Ni or azide moieties) to which cargo molecules with corresponding functionality can be conjugated (His-tagged or alkyne-functionalized proteins, respectively), B) biomolecules featuring lipidic moieties (e.g. cholesterol or alkyl chains) which can anchor the molecule into the lipid bilayer, or C) orthogonal combination of the two strategies.

Any nanoscale platform that is to be utilized as a universal vehicle for the delivery of therapeutics must be amenable to conjugation or incorporation of a diverse range of molecules featuring unique physical and chemical properties. For example, therapeutic cargo molecules can range in size, solubility, charge, and available reactive groups. Functionalizing the nanoparticle surface with reactive chemical moieties is a strategy commonly employed to achieve conjugation of therapeutic molecules and has been used extensively with great success. However, for many of these nanoparticle systems attaining functional activity on the surface of the particle is not trivial and often requires significant modifications to the particle constituents and synthesis protocols or costly additional steps post-synthesis, such as grafting the surface with a functionalized polymer [45]–[47]. Thus, most particulate systems are engineered to readily incorporate molecules of similar physical or chemical properties, rather than a host of biological molecules with highly disparate properties. However, these issues can be readily mitigated when utilizing the NLP platform because the lipid bilayer is so versatile in accommodating myriad amphipathic and lipophilic molecules. We have essentially developed two facile and generally applicable methods for conjugating biomolecules to the NLP platform. First, NLPs can be assembled with a bilayer-forming lipid (helper lipid) and functional lipids (lipids bearing a functional head group), which can be utilized for conjugation of molecules featuring the corresponding chemical reactivity (Figure 3A). Second, cargo molecules containing lipophilic groups (e.g. cholesterol) that are either native to the molecule or chemically appended through covalent attachment can be tethered to the NLP, whereby the lipophilic group anchors the compound to the NLP lipid bilayer. In this case the NLPs are assembled with the helper lipid and chemically modified biomolecule (Figure 3B). Importantly, these two broadly applicable methods can be used orthogonally to conjugate multiple biological molecules of disparate chemistries to the NLP (Figure 3C).

**Figure 4:**
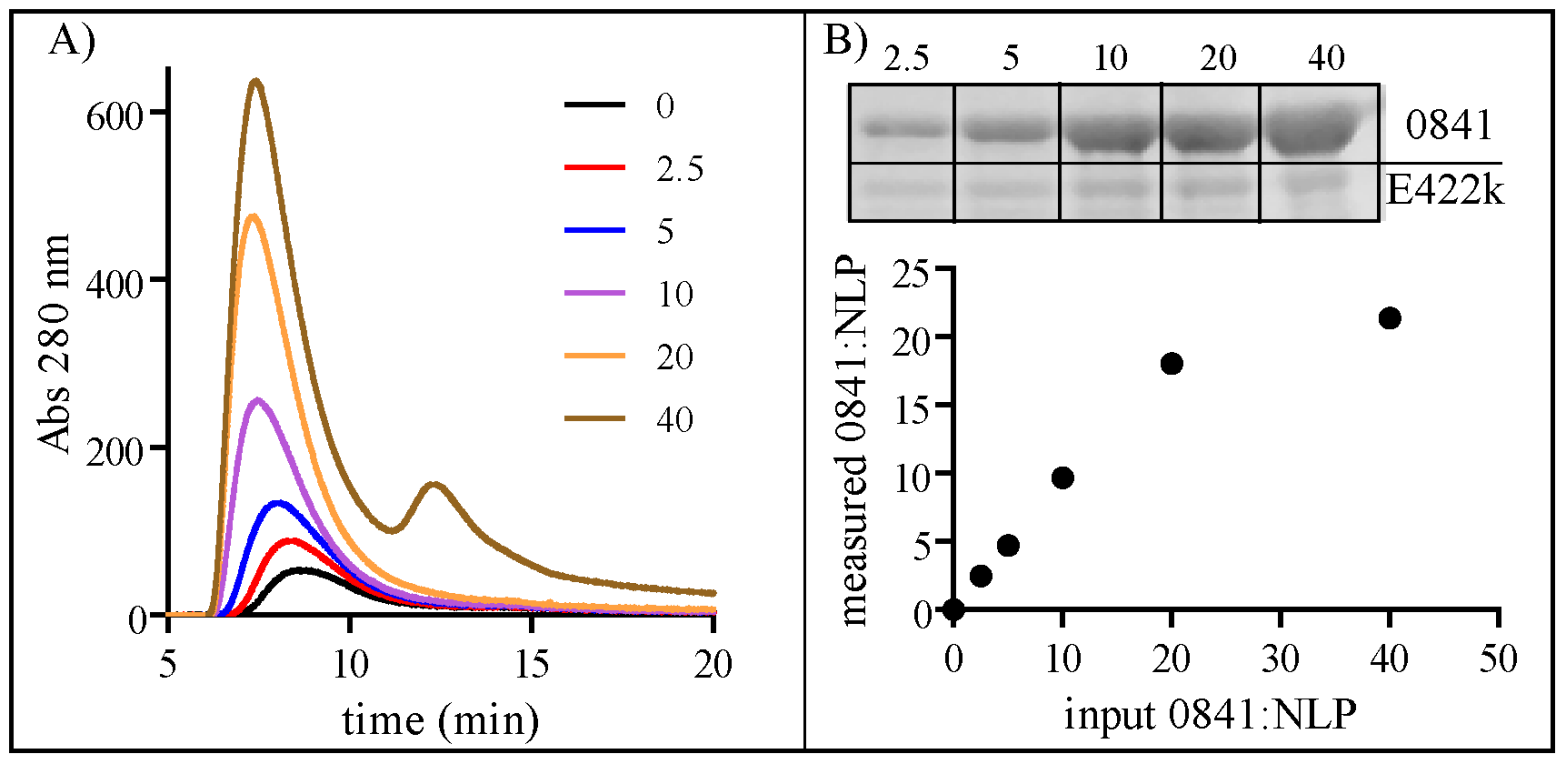
Non-covalent conjugation of protein (0841) to the NiNLP platform. A) SEC traces of NiNLPs incubated with His-tagged 0841 at indicated molar ratios. B) The 0841:NiNLPs were purified by SEC, and SDS-PAGE and densitometry were used to quantify the amount of 0841 and apoE422k protein in each NiNLP sample using known standards for each protein. These measured concentrations were used to calculate the number of proteins bound per NLP and were plotted as a function of the initial ratio used in the conjugation reaction.

To demonstrate the ease of conjugating biomolecules to a functionalized NLP lipid bilayer, strategies for both covalent and non-covalent conjugation of recombinant proteins were assessed. Each approach involved the formation of a multi-component lipid bilayer, utilizing a chemically functionalized lipid in conjunction with a non-reactive helper lipid. As discussed above, DOPC∶NLPs displayed greater stability than DMPC∶NLPs; thus, DOPC was utilized as the helper lipid in all the multifunctional NLPs described in this section. For non-covalent protein attachment, the non-covalent interaction between chelated nickel atoms and polyhistidine-tagged proteins, which has been extensively employed in both protein purification and conjugation strategies, was used. To enable conjugation of His-tagged proteins to the NLP, a commercially available nickel-chelating lipid (NiLipid) was incorporated into the NLP bilayer (referred to as NiNLPs). We have previously demonstrated that NiNLP compositions with NiLipid constituting 35% of total bilayer lipid provide the greatest conjugation efficiency in our system [37]. The purified NiNLPs were incubated with a His-tagged protein (0841 from *Francisella tularensis*) for 30 minutes and analyzed by SEC, an analytical method well suited for the characterization of biomolecule conjugation to functionalized NLPs [12], [14], [34], [37]. Free proteins typically have a t_R_ between 11.5 and 12.5 min, compared to a t_R_ ∼8–9.5 min for the larger NLP; thus, if no protein is attached to the NLP, a free protein peak should be observed at t_R_ between 11.5 and 12.5 min. The black line in Figure 4A corresponds to the NiNLP in the absence of the His-tagged protein. As expected the t_R_ of the NiNLP alone was ∼7.8 min. When His-tagged 0841 was incubated with the NiNLP at increasing 0841:NiNLP ratios prior to SEC analysis, the NLP t_R_ decreased with a concomitant increase in signal intensity (both indicators of successful cargo attachment) (Figure 4A). In addition, no free protein peak was observed at 0841:NiNLP ratios below 40∶1 (Figure 4A). These combined observations indicate that the His-tagged 0841 protein was successfully conjugated to the NiNLP platform. To verify attachment, SEC fractions corresponding to the 0841:NiNLP complex were analyzed by SDS-PAGE. As shown in Figure 4B (inset), both 0841 and apoE422k were co-localized in the NiNLP fractions, and 0841 band intensity increased commensurate with increasing ratios of 0841 to NiNLP. Densitometry of the SDS-PAGE gel bands was used to quantify the amount of 0841 immobilized on the NiNLPs at increasing concentrations, and expressed as a function of the initial reaction ratio in Figure 4B. Importantly, this relationship was almost linear with a slope of 0.91 up to a ratio of 20∶1. These results indicate that below ratios of 20 0841 molecules per NiNLP, near complete conjugation of the His-tagged protein was achieved. These combined results demonstrate that the His-tagged 0841 protein was successfully conjugated to the NiNLP platform. Importantly, we have also demonstrated successful conjugation of over 20 different His-tagged proteins to the NiNLP platform [32], [34], [37], indicating this reaction was not unique to 0841.

**Figure 5:**
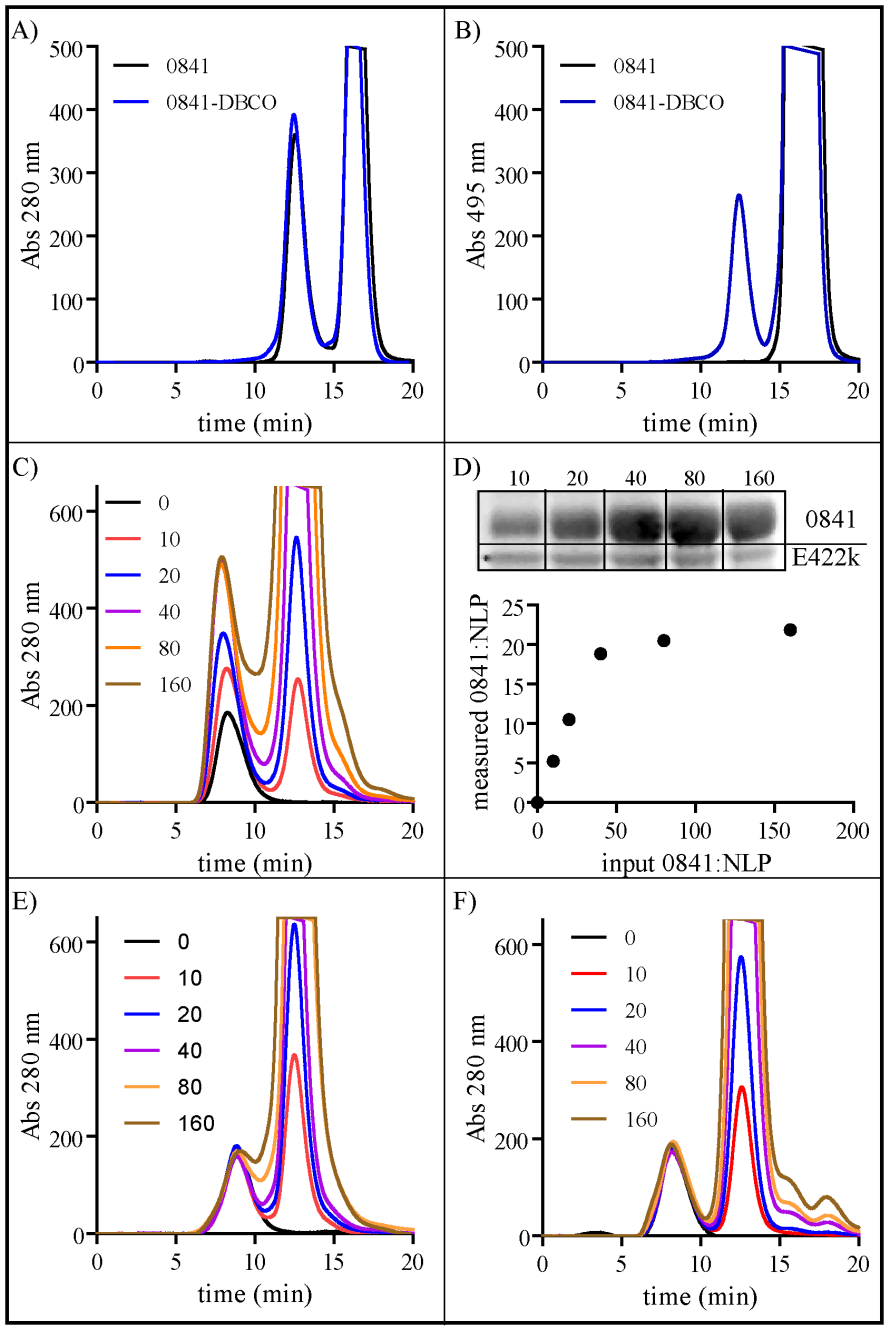
Covalent conjugation of protein (0841) to the N_3_NLPs platform. A) and B) SEC chromatograms of 0841 (black line) and 0841-DBCO (blue line) incubated with AF488-azide at a fluorescent dye to protein ratio of 5 monitored at two absorbance wavelengths: A) 280 nm and B) 495 nm. Chromatogram peaks correspond to 0841 protein (t_R_ 12.5 min) and unreacted AF488-azide (t_R_ 16.3 min). C) SEC chromatograms of N_3_NLPs incubated with 0841-DBCO at indicated molar ratios. Chromatogram peaks correspond to NLP (t_R_ 8.5 min) and unconjugated 0841 protein (t_R_ 12.5 min). D) The 0841-DBCO:N_3_NLPs complexes were purified by SEC, and SDS-PAGE and densitometry were used to quantify the amount of 0841 and apoE422k protein in each NiNLP sample using known standards for each protein. These measured concentrations were used to calculate the number of proteins bound per NLP and were plotted as a function of the initial ratio used in the conjugation reaction. E) SEC chromatograms of NLPs (lacking the C18-PEG6-N_3_) incubated with 0841-DBCO at indicated molar ratios. F) SEC chromatograms of N_3_NLPs incubated with 0841 (lacking DBCO reactive group) at indicated molar ratios.

Although the outlined non-covalent conjugation strategy was facile and highly efficient, the binding is reversible. As previously reported, the off-rates of His-tagged proteins from NiNLPs are typically less than 6 hours [37], which may not be a sufficiently long retention time for many applications; thus, a covalent conjugation strategy was employed to provide an irreversible conjugation and thereby increase the fidelity of the cargo∶NLP interaction. An important consideration for specific and tailored covalent conjugation of a biomolecule to the NLP is to ensure that chemoselective reaction pairs are used, specifically avoiding biological functional groups present on the apoE422k scaffold protein (e.g. amino and carboxyl groups). To address this issue, an azide-alkyne cycloaddition reaction (click chemistry) was investigated. Specifically, we took advantage of recent publications demonstrating strain-promoted alkyne-azide cycloaddition reactions (SPAAC) that do not require either copper catalysts or organic solvents to be effective [48]–[51]. NLPs were assembled with alkyl-modified azide moiety (C18-PEG6-N_3_), which was readily incorporated during the self-assembly reaction to form N_3_NLPs, at a molar ratio of 2.5% C18-PEG6-N_3_ and 97.5% DOPC. To achieve conjugation of a protein through click chemistry, the 0841 protein was first modified to contain an alkyne moiety by covalently coupling the NHS-activated strained alkyne molecule dibenzylcyclooctyne (DBCO) to free amines on the 0841 surface (0841-DBCO). Initially, the presence of DBCO on the surface of the 0841 protein was verified by reacting with an azide-functionalized fluorophore (AF488-azide). As demonstrated in Figure 5, AF488-azide labeled only DBCO-0841. The products from the labeling reactions were separated by SEC to resolve protein and unreacted fluorophore (t_R_ 12.5 and t_R_ 16.3, respectively). The protein absorbance was monitored at 280 nm, and the fluorophore absorbance was monitored at 495 nm. While both 0841 and 0841-DBCO had near-identical 280 nm chromatograms (Figure 5A), only 0841-DBCO (blue line) exhibited absorbance at 495 nm (Figure 5B). These results demonstrate two important points: 1) the protein was successfully functionalized with DBCO and 2) conjugation between an azide-functionalized compound and 0841-DBCO was specific.

To determine if 0841-DBCO could be covalently conjugated to the N_3_NLP, 0841-DBCO was incubated with the N_3_NLP at 0841-DBCO:N_3_NLP ratios ranging from 0 to 160 for 24 hrs, and the samples were analyzed by SEC (Figure 5C). As shown in Figure 5C, a clear shift in t_R_ and an increase in peak intensity were observed for the N_3_NLP:0841-DBCO constructs relative to the N_3_NLP alone and the magnitude of these changes increased at higher 0841-DBCO:N_3_NLP ratios, indicating successful conjugation. However, at all ratios tested, a significant peak corresponding to free 0841 was observed, indicating that the reaction was not 100% efficient. To quantify the degree of conjugation and verify that the increase in peak intensity and decrease in t_R_ were due to conjugation of the 0841-DBCO protein, the peaks corresponding to the N_3_NLP:0841-DBCO construct were analyzed by SDS-PAGE and quantified by densitometry. As shown in Figure 5D (inset), both apoE422k and 0841-DBCO were present in the NLP SEC fractions, further demonstrating that the DBCO-protein was successfully conjugated to the N_3_NLP platform. When final conjugate ratios were expressed as a function of the initial reaction ratio (Figure 5D), a linear relationship was observed up to 20 proteins per NLP, with a significantly smaller slope compared to the non-covalent approach (0.53 vs. 0.91, respectively). Based on these experimental data, a substantial amount of protein remained unreacted with the N_3_NLP, which has been widely reported with these types of strained cycloaddition reactions. Under identical experimental conditions, two control experiments were performed to verify that the observed conjugation between 0841-DBCO and N_3_NLP was due to specific chemical conjugation, rather than nonspecific interactions. First, DOPC NLPs lacking the C18-PEG6-N_3_ functional constituent were incubated with 0841-DBCO (Figure 5E). Second, N_3_NLPs were incubated with unmodified 0841 (lacking the DBCO moiety) (Figure 5F). In both control experiments, the NLP peaks in the SEC chromatograms remained unchanged, precluding the possibility that nonspecific interactions play a role in protein association with the NLP. These results indicate that conjugation occurs exclusively when both click chemistry reagents are present and that nonspecific interactions between the control components are not observed. Comprehensively, these results demonstrate our ability to successfully covalently attach a protein to the NLP platform using click chemistry, although the coupling reaction is less efficient than the comparable non-covalent approach.

**Figure 6:**
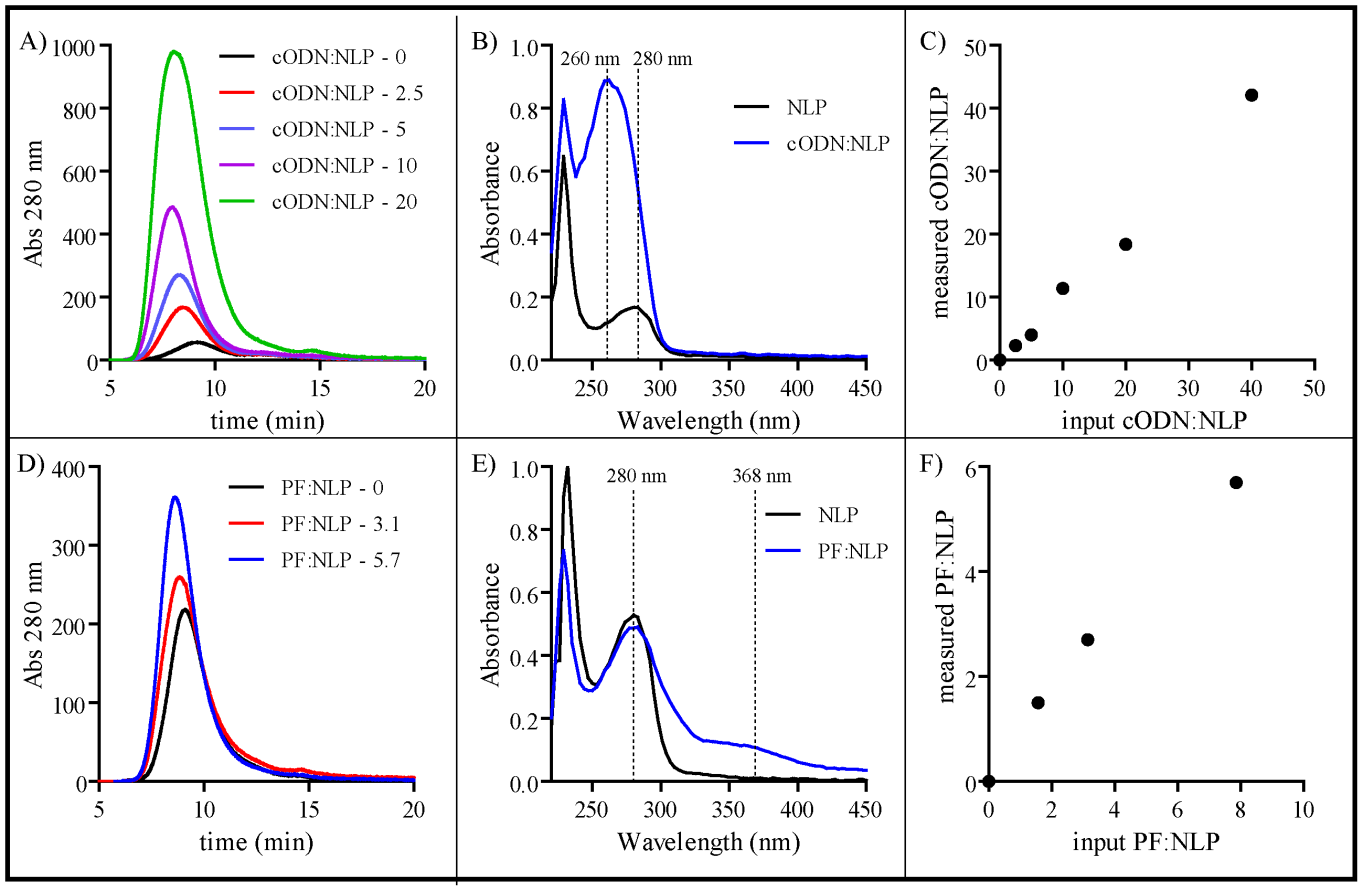
Conjugation of cODN and PF to the NLP platform. A) SEC analysis of the cODN∶NLP constructs at indicated cODN∶NLP molar ratios, monitored at 280 nm. The increase in absorbance at 280 nm and peak shift indicate successful incorporation of cODN. B) UV-Vis absorption spectra of SEC-purified cODN∶NLP (blue line) and apoE422k concentration matched NLP lacking the cODN (black line). The dashed lines represent the absorbance at 260 nm and 280 nm. C) Analysis of cODN incorporation into the NLP. The x-axis represents the cODN∶NLP ratio used during the NLP assembly reaction and the y-axis is the measured amount of cODN ultimately incorporated into the particle. D) SEC chromatograms of the PF∶NLP constructs at increasing PF-to-NLP ratios. E) UV-vis spectra of PF∶NLPs (blue line) and apoE422k concentration matched NLPs lacking the PF (black line). The dashed lines represent the absorbance at 280 nm and 368 nm. F) Analysis of PF incorporation efficiency into the NLP, represented as a function of the PF-to-NLP assembly ratio (x-axis) vs. measured PF-to-NLP ratio after purification (y-axis).

In addition to utilizing functionalized lipids for subsequent attachment of biological molecules with the corresponding chemistry, we have also developed methods to attach molecules that have been covalently modified with a lipophilic compound (Figure 6). The first compound tested was a cholesterol-modified oligodeoxynucleotide (cODN). The particular ssDNA molecule used for this study is complementary to the micro RNA mir21, an anticancer therapeutic target [52]. DOPC-based NLPs were assembled at increasing cODN∶NLP ratios and analyzed by SEC (Figure 6A). Mirroring the results seen with conjugating protein molecules to the NLPs, a decrease in t_R_ of the cODN∶NLP was concomitant with significant increases in peak absorbance intensity. Absorbance spectroscopy of purified NLPs demonstrated that only NLPs formulated with the cODNs exhibited the characteristic intrinsic absorbance of the cODN at 260 nm (Figure 6B). The absorbance of the purified cODN∶NLP was significantly higher at both 260 nm and 280 nm than control NLPs at an identical apoE422k concentration. The cODN concentration was subsequently measured for the cODN∶NLPs assembled at increasing cODN ratios (see Materials and Methods). As illustrated in Figure 6C, when the measured cODN∶NLP ratios were plotted as a function of the input cODN∶NLP ratios, a near linear trend was observed over the tested ratios (up to a cODN∶NLP ratio of 40∶1) (slope = 1.04), which indicates that incorporation of cODNs was extremely efficient.

To further demonstrate the feasibility of using this approach to attach biological molecules to the NLP platform, NLPs were assembled with DPSE-PEG2000-folate (PF) at increasing PF∶NLP ratios using DOPC as the helper lipid (Figure 6D). As was observed for the cODN∶NLP, there was a clear shift to a shorter t_R_ and an increase in the peak intensity when the NLPs were assembled with the PF (7.6 min vs. 7.8 min), both of which indicate successful conjugation. The PF∶NLP ratios of the assembled particles were analyzed based on the absorption of the purified NLPs at 368 nm, which corresponds to a unique intrinsic absorption peak of folate. As seen in Figure 6E, absorbance at 368 nm is observed only in the NLP prepared with PF. Using pure PF to derive a standard curve, absorbance values at 368 nm were then used to calculate the PF concentration in each NLP. A near linear relationship (slope = 0.71) was observed when the post-assembly PF∶NLP was plotted as a function of the input PF∶NLP (Figure 6F).

The combined results of these experiments demonstrate that the NLP platform is ideally suited for the attachment of a wide range of molecules with disparate chemical and functional properties, which is an important attribute of any nanoparticle system that is to be used for the *in vivo* delivery of therapeutic agents. Although we demonstrated the potential of attaching multiple different types of biological molecules using NLPs of varying composition and functional anchors, the NiNLP (35% NiLipid and 65% DOPC) was selected for further analysis of *in vitro* cytotoxicity, *in vivo* toxicity, *in vivo* immunogenicity and *in vivo* biodistribution as the NiNLP has been previously utilized as an *in vivo* antigen delivery vehicle for vaccine applications [32] and is one of the most versatile functional NLPs for use in delivery of any His-tagged protein [34], [37].

### In vitro cytotoxicity

**Figure 7:**
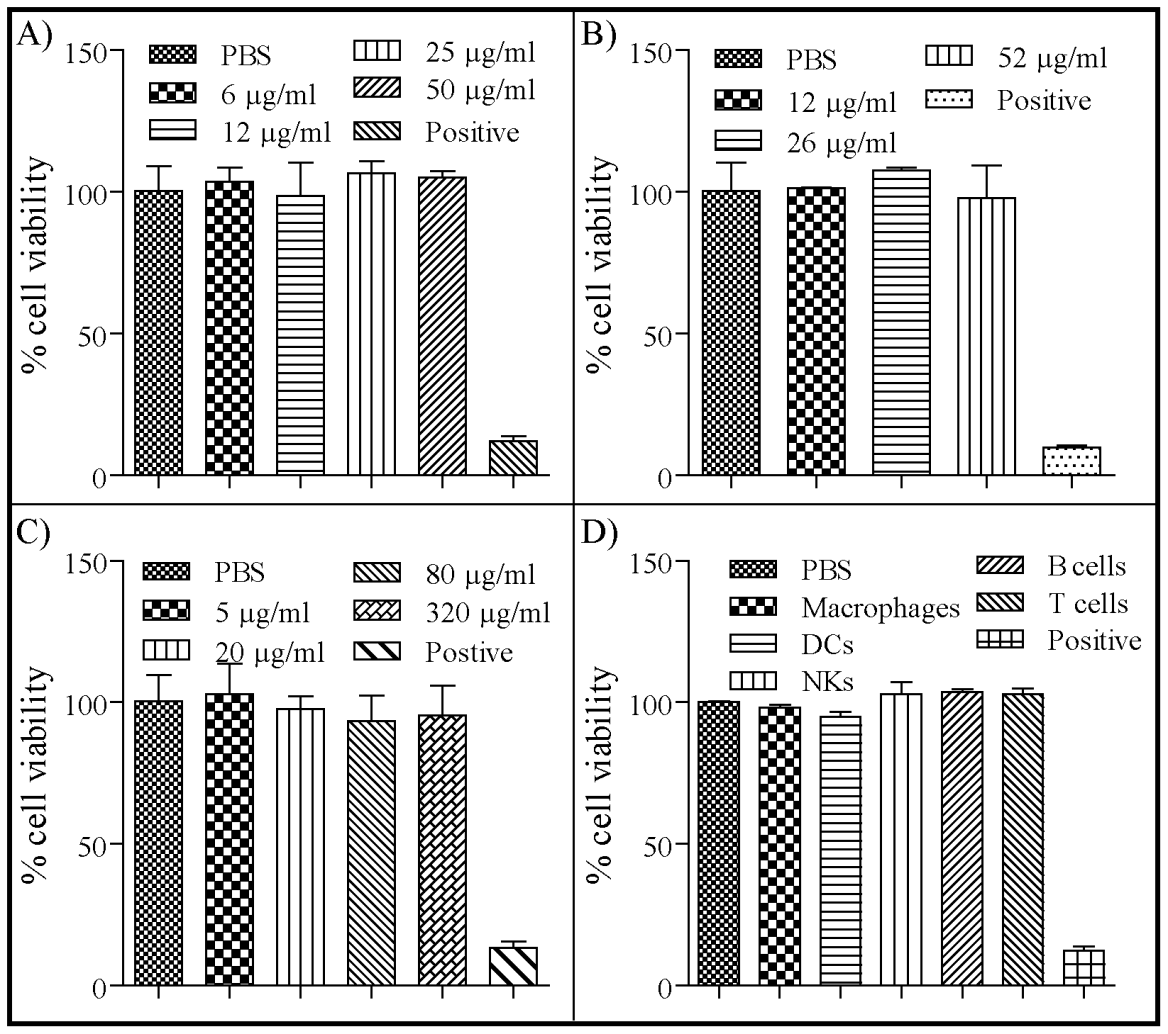
In vitro cytotoxicity of the NiNLP platform. A) NiNLP cytotoxicity was measured using the MTT assay with UMR cells at NLP treatment doses ranging from 6–50 µg/ml (cell viability values are relative to the PBS treated cells). Toxicity of the NiNLPs measured using a LDH release assay with B) J774 cells at NLP treatment doses ranging from 12–52 µg/ml, C) Hep G2 cells at NiNLP treatment doses ranging from 5–320 µg/ml, and D) primary murine immune cells (macrophages, DCs, NKs, B cells, and T cells) at an NiNLP dose of 25 µg/ml. All values in the LDH release studies were normalized to the PBS treated cells. Lysed cells were used as a positive control. Results are shown as mean values from duplicate experiments, with error bars representing standard deviation values.

Cytotoxicity has been documented for metal-based nanoparticles [53], [54] and other non-biological based nanoparticles, such as dendrimers [55]. These types of nanoparticle platforms often require complex modifications to limit their cytotoxic effects. In contrast, the NLP platform consists solely of biocompatible components and is in fact a mimic of nanoparticles that are present in the human body (HDLs); thus, we hypothesized that the cytotoxic effect of NLPs would be minimal. To address this question, the *in vitro* cytotoxicity of the NiNLP was evaluated. These experiments were performed using UMR-106 (rat osteosarcoma) cells, J774 (mouse macrophage cell line) cells, HEP-G2 (human hepatic carcinoma) cells, and a number of primary mouse splenocytes sorted by specific cell type (macrophages), dendritic cells (DCs), natural killer (NK) cells, B cells, and T cells. The NiNLPs displayed no cytotoxic effects when incubated with the UMR-106 cells at concentrations ranging from 1.6–50 µg/ml (based on amount of apoE422k in the sample; data was normalized to the PBS control) as assessed using the MTT assay (Figure 7A). Similarly, no toxicity was observed in J774 cells when incubated with NiNLPs at concentrations ranging from 0–52 µg/ml (based on amount of apoE422k in the sample; data was normalized relative to the PBS control and positive cells correspond to cells that were lysed by multiple freeze-thaw cycles) when determined using a lactate dehydrogenase (LDH) activity assay (Figure 7B). To further demonstrate the low cytotoxicity of the NiNLP platform, these experiments were repeated with a human liver cell line, Hep G2. This cell line has been widely used as a model cell line to assess potential cytotoxicity of drugs in the liver [56]. In these experiments, cells were incubated with up to 320 µg/ml of the NiNLP for 24 hrs and cytotoxicity was measured using the LDH activity assay (Figure 7C). As observed with the other cell types, the NiNLPs displayed no significant cytotoxicity relative to the PBS control. Based on these initial results, we proceeded to evaluate the cytotoxicity of 25 µg/ml of NiNLP against a variety of primary mouse immune cell types isolated from the spleen (Figure 7D). At this dose, no cytotoxicity was observed in any cell type examined. Thus, these combined data indicate that the NiNLP platform displays no cytotoxicity *in vitro*, even at concentrations as high as 320 µg/ml, which is likely due in part to the biocompatibility of the NLP composition. It is worth noting that only the NiNLP platform was evaluated in these studies; thus, the low cytotoxicity observed here cannot be generalized to the other compositions described in the multifunctional NLP section. However, since DOPC was the primary lipid constituent in the NiNLP and the other NLP compositions described above, it is predicted that these compositions would also display a low cytotoxicity *in vitro*.

### In vivo acute toxicity

**Figure 8:**
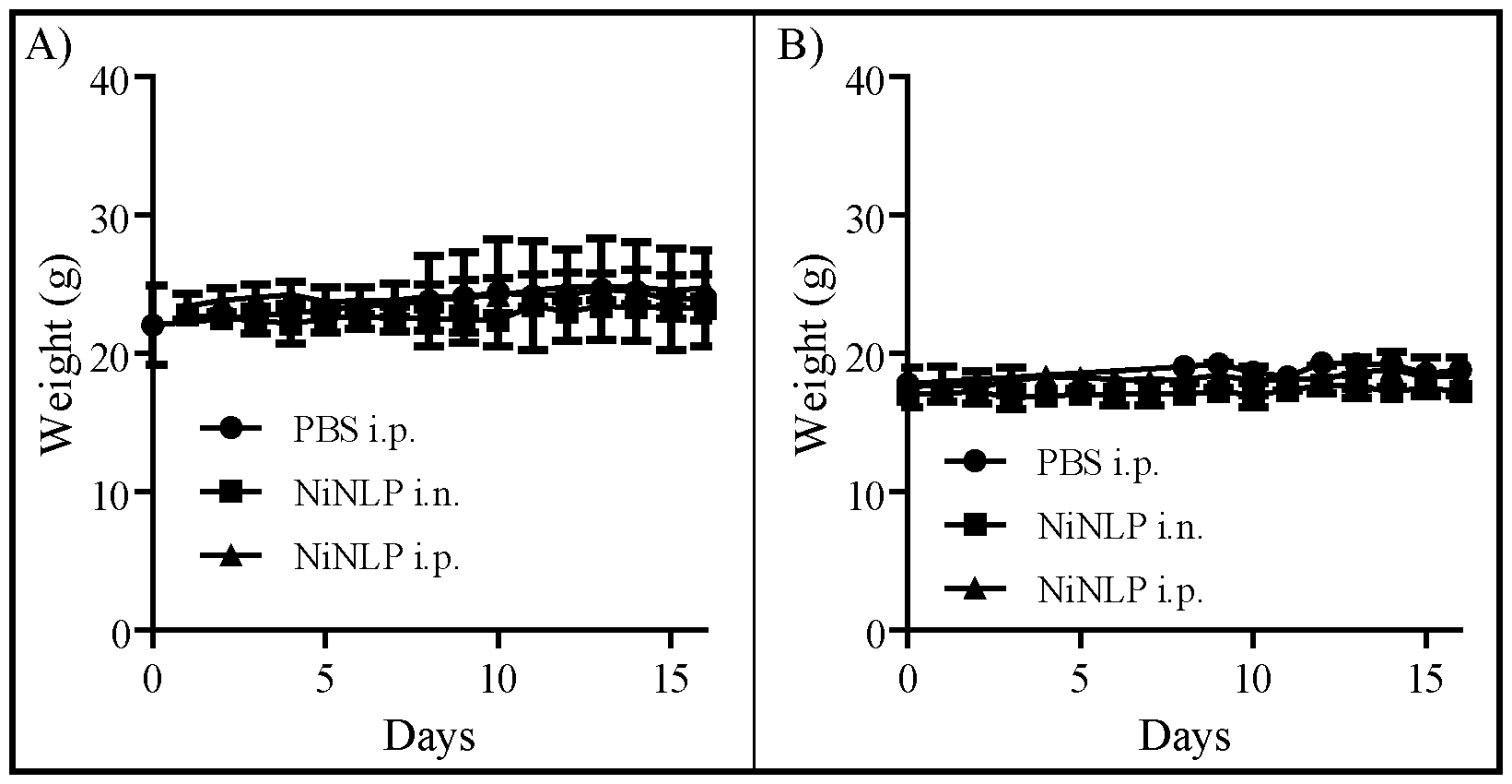
Effect of repeated NiNLP administration on mouse body weights. Weights of A) male and B) female mice receiving daily NiNLP injections i.n. (30 µl) or i.p. (100 µl) for 14 consecutive days. Control mice received equal volumes of PBS i.p. over the same 14-day time course. Data represent averaged weights from groups of three animals, with standard deviation error bars.

**Figure 9:**
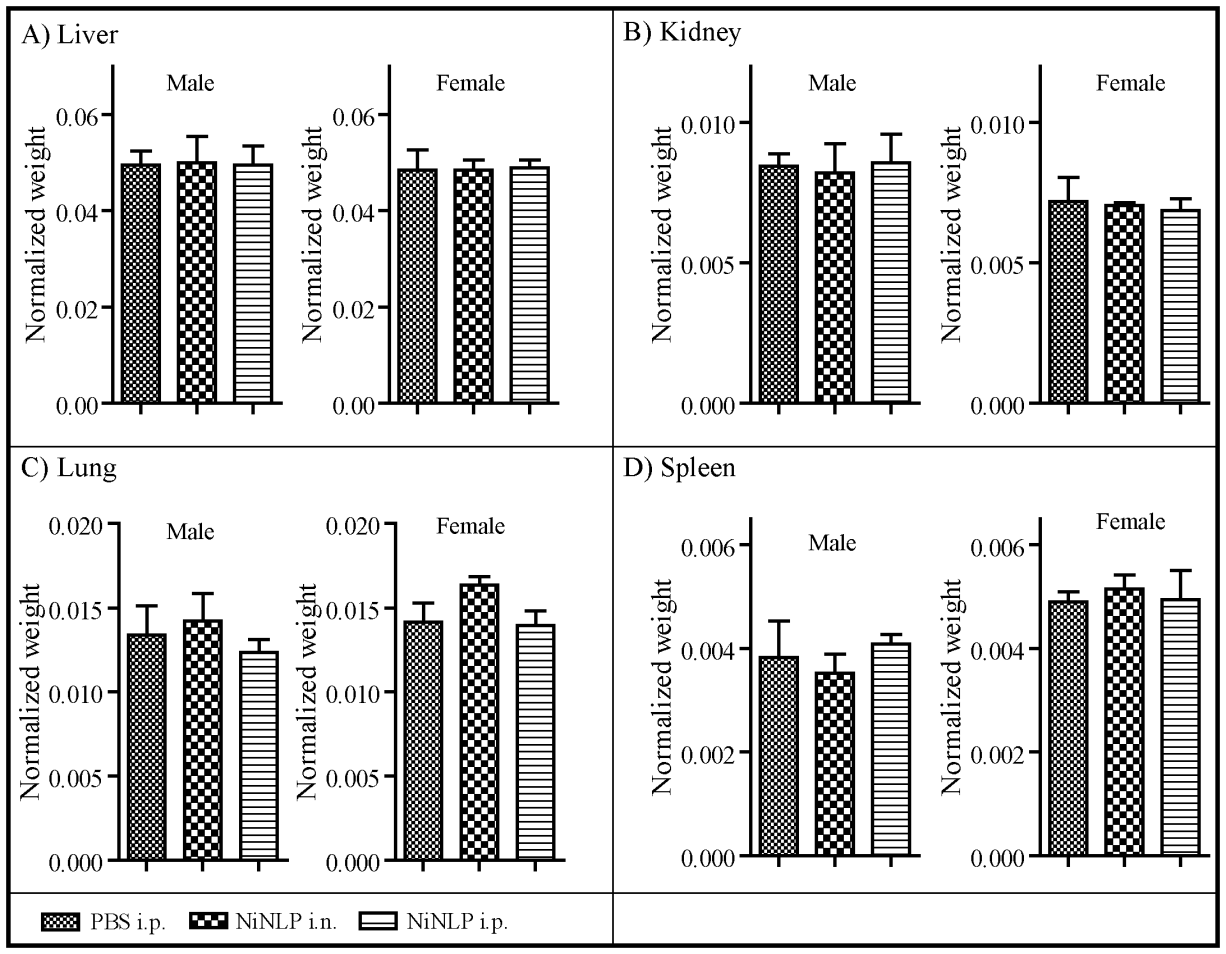
Effect of repeated NiNLP administration on mouse organ weights. Weights of A) liver, B) kidney, C) lung, and D) spleen obtained from mice that received 25 µg of NiNLP i.n. (30 µl) or i.p. (100 µl) daily for 14 consecutive days. Control animals received an equal volume of PBS i.p.(100 µl) daily for 14 days. Normalized organ weights are represented as (organ weight, g)/(body weight, g). Data represent averaged organ weights from groups of three animals, with standard deviation error bars.

Next, we set out to determine whether NLPs were also non-toxic *in vivo*. As described above, these experiments were performed using the NiNLP construct. Acute toxicity experiments were performed according to the National Toxicology Program published by the Department of Health and Human Services (http://ntp.niehs.nih.gov/). In these experiments, groups of six mice (3 male/3 female) were injected with 25 µg of NiNLP (based on apoE422k content) either through the i.p. or i.n. route daily for 14 consecutive days. The 25 µg dose was selected based on typical doses that have been previously used in NiNLP-based vaccine formulations [30], [32]. Animals were weighed daily and assessed for overt signs of morbidity. As shown in Figure 8, no significant weight loss was observed in either males (Figure 8A) or females (Figure 8B) over the course of 14 days relative to PBS control animals, indicating that daily administration of the NiNLP over this time period did not result in any overt signs of animal distress or diminished health. Similarly, no differences in responsiveness and activity level of the animals were observed between the groups. At the end of the 14 day time period, the mice were euthanized, and liver, lung, kidney and spleen were collected, weighed, and visually inspected for any signs of lesions and obvious tissue damage. No obvious lesions were observed in any of the harvested tissues (data not shown), and no statistically significant differences were observed in normalized weights of the organs (organ weight/mouse weight) between the PBS group and the mice that received the NiNLP (Figure 9).

**Figure 10:**
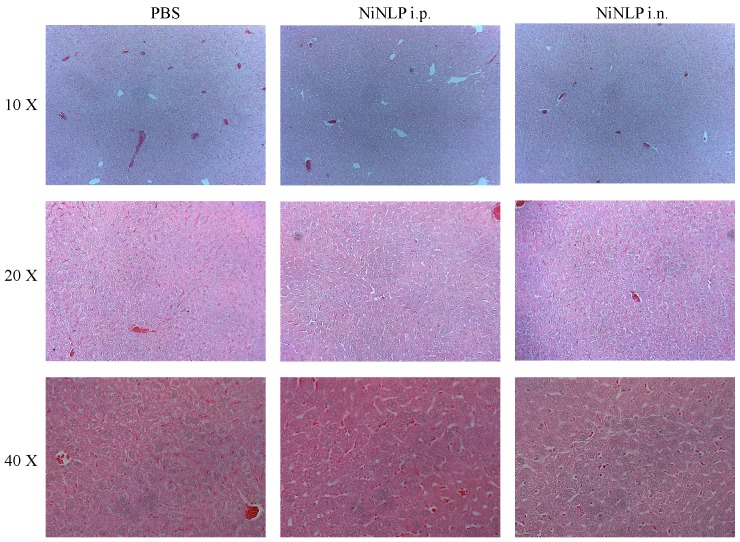
Histological analysis of the effect of repeated NiNLP administration on the microstructure of the liver. H&E stained sections of livers obtained from mice that had received 25 µg of NiNLP i.n. (30 µl) or i.p. (100 µl) daily for 14 consecutive days at 10×, 20× and 40× magnifications.

Although we observed no overt signs of damage to the organs, it is possible that daily administration of the NiNLPs had an effect on the microstructure of these organs. Therefore, the excised organs were fixed, sectioned, stained with H&E and imaged to evaluate any effect the NiNLPs may have had on the microstructure. Figure 10 shows images of H&E stained sections of the liver from mice that received PBS and NiNLPs via the i.p and i.n. route at 10×, 20× and 40× magnification. As shown in these images no obvious damage to the microstructure of the liver was observed, a trend similarly observed for the spleen, kidney and lung (data not shown). These combined results demonstrate that the NiNLP platform was biocompatible and displayed no acute toxicity *in vivo* when administered daily over a two week period.

The low toxicity of the NiNLPs was not surprising given that the particles are essentially a mimetic of naturally occurring particles, HDLs. Therefore, we hypothesize that by utilizing a platform that mimics naturally occurring particles, we have significantly reduced any potential toxic effects that would be associated with the delivery vehicle itself. It is worth noting that HDLs are produced and processed in the liver, underscoring the possibility that this aggressive administration protocol could have caused adverse effects in this organ; however, this was not observed. Typical serum concentrations of HDLs in the mouse are approximately 0.150 mg/mL [57], although this value can greatly fluctuate daily depending on diet. As such, the daily dose of 25 µg constituted only ∼10% of total serum HDL levels (assuming an average blood volume of approximately 1.5 ml for a 25 g mouse and average HDL concentration of 0.15 mg/mL), which is well within a tolerable HDL range.

These experiments were performed with only the NiNLP platform and the low acute toxicity observed in these studies cannot be generalized to all NLP compositions; however, since the primary lipid constituent was DOPC, it is likely that other DOPC-based NLPs would also display a similarly low toxicity.

### In vivo immunogenic properties

**Figure 11:**
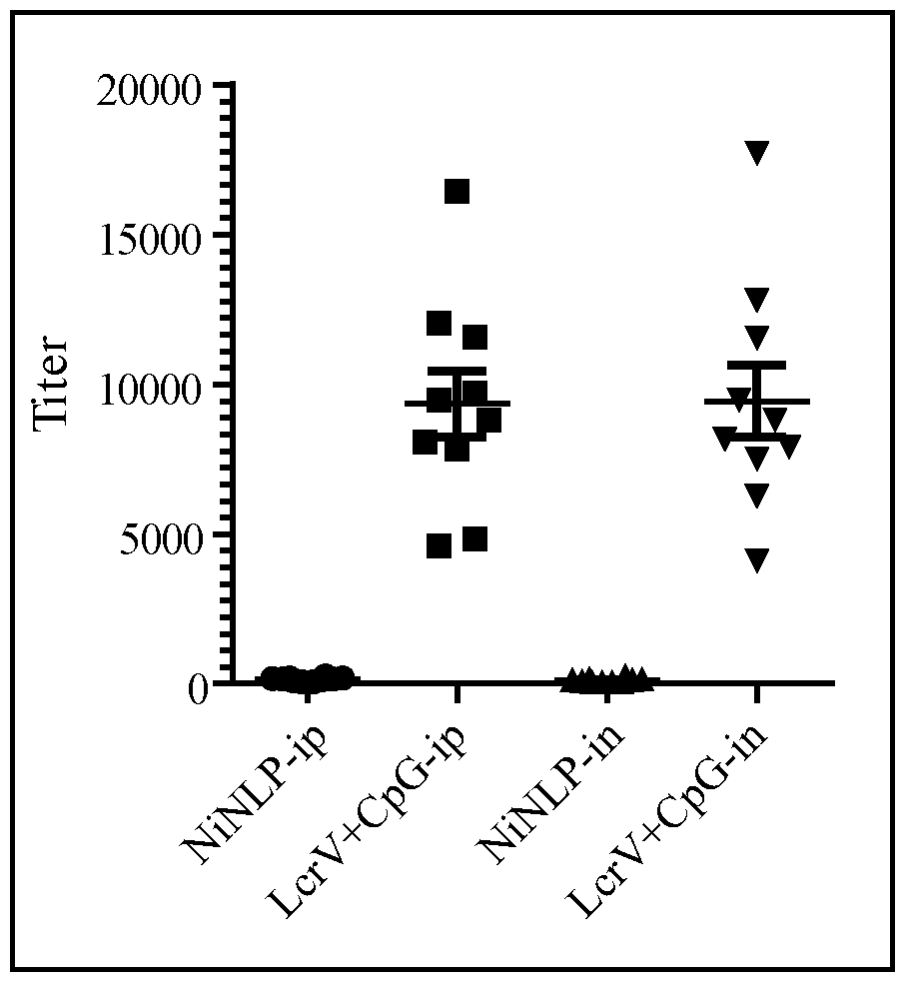
Assessment of NiNLP immunogenicity. Groups of 10 female BALB/c mice were inoculated either i.n. or i.p. with NiNLP. As a positive control, a group of mice was injected with a known immunogenic recombinant subunit antigen (LcrV) co-administered with adjuvant (CpG). Serum IgG antibody titers against the scaffold protein, apoE422k (NiNLP-ip and NiNLP-in), or LcrV (LcrV+CpG-ip and LcrV+CpG-in) were assessed 4 weeks post-immunization. Each data point represents the titer value of an individual mouse.

One of the primary drawbacks of several nanoparticle-based delivery vehicles is unintended immunogenic properties, which can cause unnecessary inflammatory side effects or result in rapid clearance of the particle by the immune system [58], [59]. The origin of the immunogenicity of many of these particles is due to their foreign nature, i.e. the body recognizes the particle as non-self. However, because the NLP platform is a mimetic of naturally occurring HDLs, it is possible that it will be very weakly immunogenic or non-immunogenic. Therefore, the immunogenic properties of the NiNLP platform were evaluated *in vivo* by measuring antibody generation against the apoE422k scaffold protein. In these experiments, groups of 10 mice were immunized via i.p. and i.n. administration with the NiNLP. To provide a comparative control sample known to elicit significant antibody titers, the *Y. pestis* antigen, LcrV, co-administered with the adjuvant CpG, was used as a positive control. Four weeks post-immunization, apoE422k- (NiNLP scaffold protein) (mice immunized with NiNLP) and LcrV-specific IgG antibody titers (mice immunized with LcrV+CpG) were quantified by ELISA. Significant antibody generation was observed for the positive control, LcrV+CpG, whereas almost no antibody generation against the scaffold protein used to make the NiNLP was observed for either route of administration (Figure 11). It is worth noting that LcrV+CpG was chosen as a positive control because this is an established immunogenic formulation [30]. These results demonstrate that the NiNLP platform in the absence of a cargo molecule has non-significant immunogenic properties, which is an important attribute of any drug delivery vehicle.

### In vivo biodistribution

**Figure 12:**
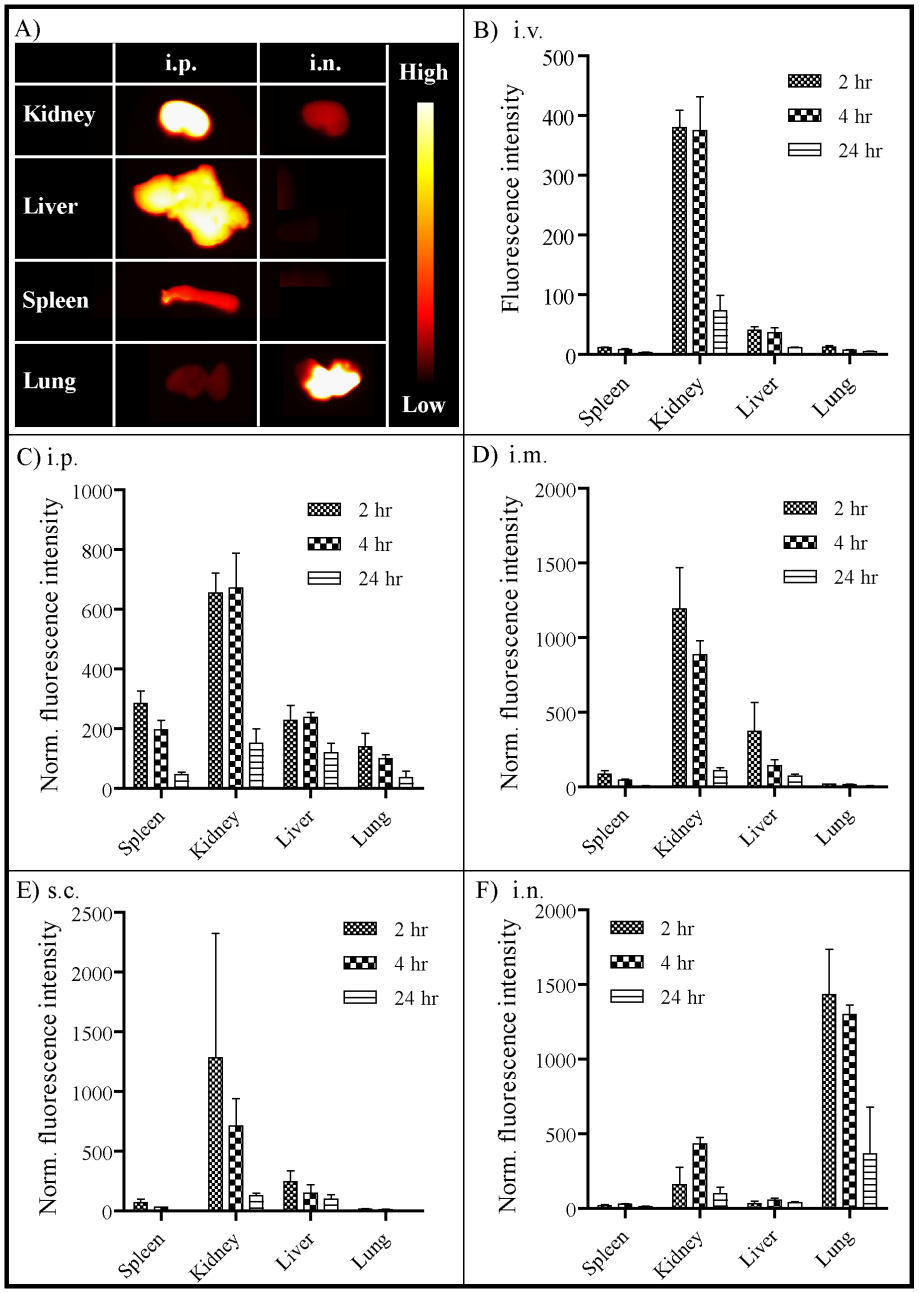
*In vivo* biodistribution of the NiNLP. A) Fluorescent images of the kidney, liver, spleen and lung 2 hrs after i.p. and i.n administration of AF750-labeled NiNLPs. Groups of 3 mice were injected B) i.v., C) i.p., D) i.m., E) s.c. or F) i.n. with AF750-labeled NiNLPs. Organs were excised 2, 4, and 24 hrs post-injection and fluorescence intensity quantified as a function of time and normalized to total organ weight. Data represent the average normalized fluorescence from groups of three animals, with standard error bars.

Successful *in vivo* delivery of therapeutics using nanoparticle-based approaches requires sustained retention of the particles *in vivo* for maximal efficacy. To evaluate the biodistribution of the NiNLP construct *in vivo*, NiNLPs were labeled with AF750 and administered i.v., i.p., i.m., s.c. and i.n. Various time points after administration of the fluorescent NLPs (2, 4 and 24 hrs), the organs were excised and imaged. Examples of fluorescent images of the kidney, liver, spleen and lung 2 hrs after i.p. and i.n. injection are shown in Figure 12A. The fluorescence intensities of organs were then measured based on these images (background fluorescence of each organ was determined based on mice that received a PBS injection) and normalized to total organ weight. When the NiNLPs were administered via the i.v. route, a robust signal was observed primarily in the kidney up to 4 hrs after injection and a smaller signal was observed in the liver (Figure 12B). This was not unexpected since the kidney primarily functions to filter the blood. In contrast, administration of fluorescent NiNLPs i.p. resulted in peak fluorescence intensities in the kidney, liver and spleen 2–4 hrs (Figure 12C) post-administration with most fluorescent signals diminished beyond 24 hrs post NiNLP administration. A similar trend was observed after i.m. and s.c. administration (Figure 12D–E), with the exception that the fluorescence intensity in the spleens were significantly lower when compared to i.p. administration. However, i.n. administration resulted in a much different biodistrubtion profile, where the majority of the signal was observed in the lung over the 24 hr time period, and to a lesser extent in the kidney (Figure 12F). These results imply that after i.n. administration the NiNLP platform is retained primarily in the lung and eventually processed by the kidney, which likely occurs through blood filtration. These results have important implications with regards to therapeutic delivery to the lungs. The lung environment has become an important target for the delivery of therapeutics using nanoparticles due to the large alveolar surface area suitable for drug absorption, the low thickness of the epithelial barrier, extensive vascularization, relatively low proteolytic activity in the alveolar space when compared to other routes of administration, and the lack of first-pass metabolism [60]–[62]. In addition, it has also been reported that uptake of nanoparticles by alveolar macrophages is reduced with particle sizes below 260 nm [63]. The significantly higher retention observed in the lung compared to the other routes of administration suggests that this delivery route may be ideal for *in vivo* delivery of therapeutics using the NiNLP platform.

**Figure 13:**
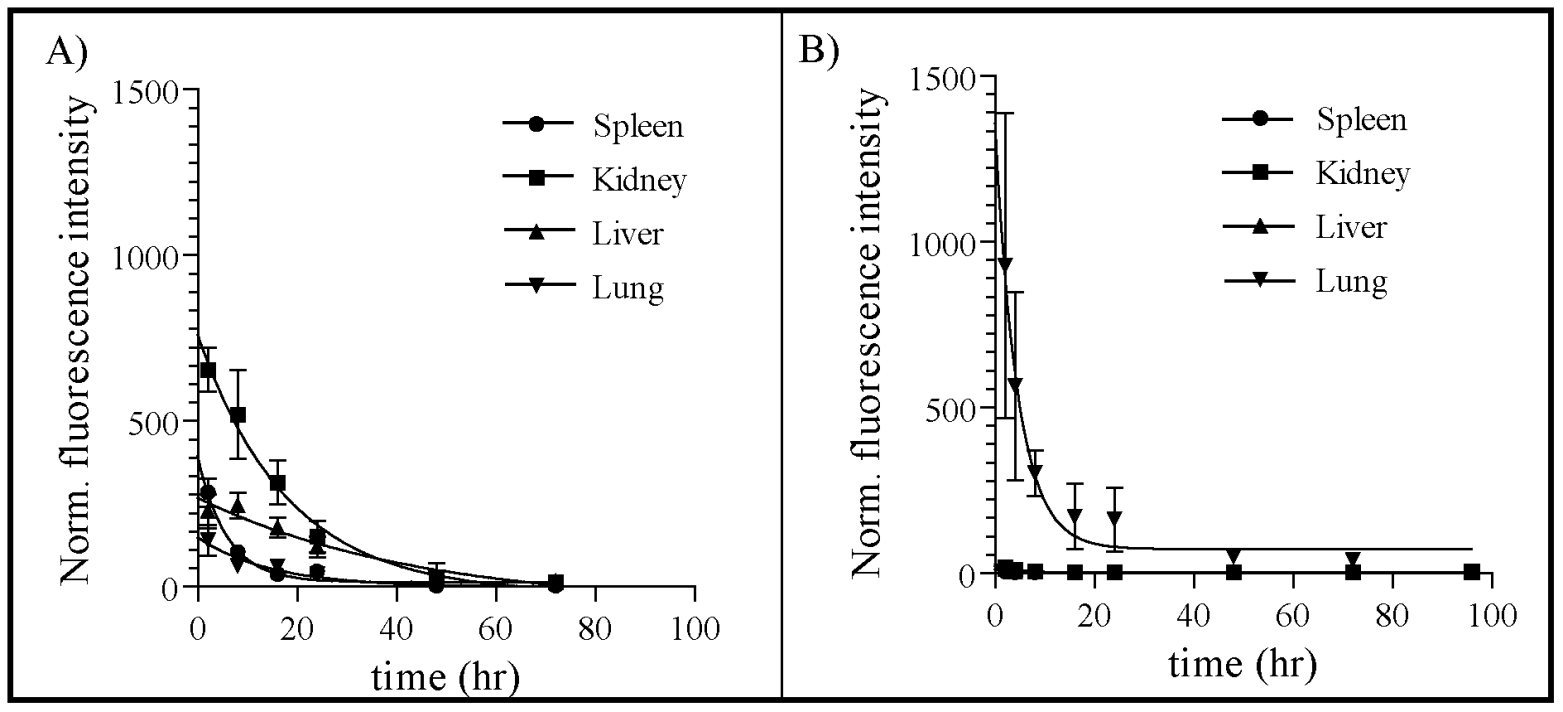
Time-dependent *in vivo* NiNLP biodistribution upon i.p. and i.n. administration. NiNLPs were administered by A) i.p. or B) i.n. routes and were assessed over 72 or 96 hours, respectively. Organ fluorescence was determined *ex vivo* and normalized to total organ weight. The normalized fluorescent intensity was quantitatively measured as a function of time. Data represent the average normalized fluorescence from groups of two animals, with standard error bars.

Due to the significant differences in the biodistribution profiles of i.n and all other routes of administration tested, an in-depth time course analysis of NiNLP biodistribution was performed for the i.n. and i.p. routes (Figure 13). Mirroring the previous study, the fluorescence signal of NLPs administered i.p. was dispersed throughout the kidney and liver, with very little fluorescence observed after 48 hrs. With i.n. administration, however, the majority of fluorescence was observed localized in the lung at all measured time points. Interestingly, the overall half-life of the NiNLP after i.p. administration (∼15 hr) was longer than what was observed after i.n. administration (∼5 hr), even though some NiNLP signal was evident over a longer time period in the lung than the other tissues after i.n. administration. These findings suggest that clearance through the lung occurs by a different mechanism after i.n. administration than was observed after i.p. administration. It is worth noting that these experiments were performed by labeling the apoE422k protein, and as such, the data do not necessarily indicate clearance of the intact particle. We are currently evaluating the biodistribution of the intact particle versus the lipid and apoE422k components by fluorescence resonance energy transfer (FRET). While the biodistribution of specific NLP formulations will be greatly impacted by their respective compositions (e.g. cargo and/or targeting molecules, surface charge), these results provide important baseline parameters of NLP biodistribution to inform and guide future studies.

## Conclusions

Despite the significant advantages that nanoparticle delivery vehicles have been shown to provide relative to conventional drug delivery systems, numerous limitations need to be addressed to increase the efficacy and broad application of nanoparticulate platforms for delivering therapeutic cargo molecules, including facile functionalization of the platform, *in vivo* stability, targeting specificity, drug entrapment efficiency, long term storage, and toxicity. Here, we evaluated NLP stability in complex biological fluids and the potential of conjugating a diverse range of biological molecules with disparate chemistries onto the NLP. In addition, the toxicity (*in vitro* and *in vivo*), immunogenicity and biodistribution of the NiNLP platform were evaluated. In these studies, the DOPC∶NLP was found to be relatively stable in complex biological fluids, with a half-life at 37°C as high as 7.5 hrs in 20% sera and 15 min in 100% sera. In addition, the amphipathic nature of the NLP platform provided facile conjugation of myriad molecules through either surface conjugation or lipidic anchoring strategies, approaches that can readily be adapted for orthogonal functionalization schemes. Accordingly, we demonstrated successful conjugation of a wide range of different molecules on the NLP, including non-covalent and covalent attachment of proteins as well as incorporation of ssDNA and a small molecule (folate). Furthermore, the NiNLP platform, which was selected for further analysis due to its potential use in vaccine applications, was nontoxic *in vitro*, and both nontoxic and nonimmunogenic *in vivo*. Finally, the biodistribution of the NiNLP was found to be highly dependent on the route of administration, where i.n. administration resulted in prolonged retention in the lung tissue and clearance through the kidney and liver was observed after i.v., i.p., i.m. and s.c. administration. The combined results of this study suggest that the NLP platform, particularly the NiNLP construct, may be ideally suited for use in both passive and targeted *in vivo* delivery of a wide range of therapeutics molecules.

## References

[1] Cho K, Wang X, Nie S, Chen ZG, Shin DM (2008) Therapeutic nanoparticles for drug delivery in cancer. Clin Cancer Res 14: 1310–1316. 18316549 10.1158/1078-0432.CCR-07-1441

[2] Mareeva T, Wanjalla C, Schnell MJ, Sykulev Y (2010) A novel composite immunotoxin that suppresses rabies virus production by the infected cells. J Immunol Methods 353: 78–86. 19932697 10.1016/j.jim.2009.11.010 PMC2823984

[3] Bhatt R, de Vries P, Tulinsky J, Bellamy G, Baker B, et al (2003) Synthesis and in vivo antitumor activity of poly(l-glutamic acid) conjugates of 20S-camptothecin. J Med Chem 46: 190–193. 12502373 10.1021/jm020022r

[4] Kim TY, Kim DW, Chung JY, Shin SG, Kim SC, et al (2004) Phase I and pharmacokinetic study of Genexol-PM, a cremophor-free, polymeric micelle-formulated paclitaxel, in patients with advanced malignancies. Clin Cancer Res 10: 3708–3716. 15173077 10.1158/1078-0432.CCR-03-0655

[5] Malik N, Evagorou EG, Duncan R (1999) Dendrimer-platinate: a novel approach to cancer chemotherapy. Anticancer Drugs 10: 767–776. 10573209

[6] Markman M (2006) Pegylated liposomal doxorubicin in the treatment of cancers of the breast and ovary. Expert Opin Pharmacother 7: 1469–1474. 16859430 10.1517/14656566.7.11.1469

[7] Manchester M, Singh P (2006) Virus-based nanoparticles (VNPs): platform technologies for diagnostic imaging. Adv Drug Deliv Rev 58: 1505–1522. 17118484 10.1016/j.addr.2006.09.014

[8] Wu W, Wieckowski S, Pastorin G, Benincasa M, Klumpp C, et al (2005) Targeted delivery of amphotericin B to cells by using functionalized carbon nanotubes. Angew Chem Int Ed Engl 44: 6358–6362. 16138384 10.1002/anie.200501613

[9] Cuenca AG, Jiang H, Hochwald SN, Delano M, Cance WG, et al (2006) Emerging implications of nanotechnology on cancer diagnostics and therapeutics. Cancer 107: 459–466. 16795065 10.1002/cncr.22035

[10] Chapman MJ (1980) Animal lipoproteins: chemistry, structure, and comparative aspects. J Lipid Res 21: 789–853. 7003040

[11] Blanchette CD, Law R, Benner WH, Pesavento JB, Cappuccio JA, et al (2008) Quantifying size distributions of nanolipoprotein particles with single-particle analysis and molecular dynamic simulations. Journal of Lipid Research 49: 1420–1430. 18403317 10.1194/jlr.M700586-JLR200

[12] Chromy BA, Arroyo E, Blanchette CD, Bench G, Benner H, et al (2007) Different apolipoproteins impact nanolipoprotein particle formation. Journal of the American Chemical Society 129: 14348–14354. 17963384 10.1021/ja074753y

[13] Miyazaki M, Tajima Y, Ishihama Y, Handa T, Nakano M (2013) Effect of phospholipid composition on discoidal HDL formation. Biochim Biophys Acta 1828: 1340–1346. 23357357 10.1016/j.bbamem.2013.01.012

[14] Fischer NO, Blanchette CD, Segelke BW, Corzett M, Chromy BA, et al (2010) Isolation, characterization, and stability of discretely-sized nanolipoprotein particles assembled with apolipophorin-III. PLoS One 5: e11643. 20657844 10.1371/journal.pone.0011643 PMC2906516

[15] Ma CI, Beckstead JA, Thompson A, Hafiane A, Wang RH, et al (2012) Tweaking the cholesterol efflux capacity of reconstituted HDL. Biochem Cell Biol 90: 636–645. 22607224 10.1139/o2012-015 PMC3697153

[16] Toledo JD, Cabaleiro LV, Garda HA, Gonzalez MC (2012) Effect of reconstituted discoidal high-density lipoproteins on lipid mobilization in RAW 264.7 and CHOK1 cells. J Cell Biochem 113: 1208–1216. 22095661 10.1002/jcb.23453

[17] Hoang A, Drew BG, Low H, Remaley AT, Nestel P, et al (2012) Mechanism of cholesterol efflux in humans after infusion of reconstituted high-density lipoprotein. Eur Heart J 33: 657–665. 21498847 10.1093/eurheartj/ehr103 PMC6590868

[18] Nestler JE, Bamberger M, Rothblat GH, Strauss JF 3rd (1985) Metabolism of high density lipoproteins reconstituted with [3H]cholesteryl ester and [14C]cholesterol in the rat, with special reference to the ovary. Endocrinology 117: 502–510. 4017944 10.1210/endo-117-2-502

[19] Baylon JL, Lenov IL, Sligar SG, Tajkhorshid E (2013) Characterizing the Membrane-Bound State of Cytochrome P450 3A4: Structure, Depth of Insertion, and Orientation. J Am Chem Soc 135: 8542–8551. 23697766 10.1021/ja4003525 PMC3682445

[20] Justesen BH, Laursen T, Weber G, Fuglsang AT, Moller BL, et al (2013) Isolation of monodisperse nanodisc-reconstituted membrane proteins using free flow electrophoresis. Anal Chem 85: 3497–3500. 23458128 10.1021/ac4000915

[21] Wadsater M, Laursen T, Singha A, Hatzakis NS, Stamou D, et al (2012) Monitoring shifts in the conformation equilibrium of the membrane protein cytochrome P450 reductase (POR) in nanodiscs. J Biol Chem 287: 34596–34603. 22891242 10.1074/jbc.M112.400085 PMC3464565

[22] Blanchette CD, Cappuccio JA, Kuhn EA, Segelke BW, Benner WH, et al (2009) Atomic force microscopy differentiates discrete size distributions between membrane protein containing and empty nanolipoprotein particles. Biochim Biophys Acta 1788: 724–731. 19109924 10.1016/j.bbamem.2008.11.019

[23] Cappuccio JA, Blanchette CD, Sulchek TA, Arroyo ES, Kralj JM, et al (2008) Cell-free co-expression of functional membrane proteins and apolipoprotein, forming soluble nanolipoprotein particles. Mol Cell Proteomics 7: 2246–2253. 18603642 10.1074/mcp.M800191-MCP200 PMC2577204

[24] Cappuccio JA, Hinz AK, Kuhn EA, Fletcher JE, Arroyo ES, et al (2009) Cell-free expression for nanolipoprotein particles: building a high-throughput membrane protein solubility platform. Methods Mol Biol 498: 273–296. 18988032 10.1007/978-1-59745-196-3_18

[25] Tufteland M, Ren G, Ryan RO (2008) Nanodisks derived from amphotericin B lipid complex. J Pharm Sci 97: 4425–4432. 18271034 10.1002/jps.21325 PMC2893587

[26] Yuan Y, Wang W, Wang B, Zhu H, Zhang B, et al (2013) Delivery of hydrophilic drug doxorubicin hydrochloride-targeted liver using apoAI as carrier. J Drug Target 21: 367–374. 23600747 10.3109/1061186X.2012.757769

[27] Ding Y, Wang W, Feng M, Wang Y, Zhou J, et al (2012) A biomimetic nanovector-mediated targeted cholesterol-conjugated siRNA delivery for tumor gene therapy. Biomaterials 33: 8893–8905. 22979990 10.1016/j.biomaterials.2012.08.057

[28] Gaidukov L, Bar D, Yacobson S, Naftali E, Kaufman O, et al (2009) In vivo administration of BL-3050: highly stable engineered PON1-HDL complexes. BMC Clin Pharmacol 9: 18. 19922610 10.1186/1472-6904-9-18 PMC2785756

[29] Frias JC, Ma Y, Williams KJ, Fayad ZA, Fisher EA (2006) Properties of a versatile nanoparticle platform contrast agent to image and characterize atherosclerotic plaques by magnetic resonance imaging. Nano Lett 6: 2220–2224. 17034087 10.1021/nl061498r

[30] Fischer NO, Rasley A, Corzett M, Hwang MH, Hoeprich PD, et al (2013) Colocalized delivery of adjuvant and antigen using nanolipoprotein particles enhances the immune response to recombinant antigens. J Am Chem Soc 135: 2044–2047. 23331082 10.1021/ja3063293

[31] Bhattacharya P, Grimme S, Ganesh B, Gopisetty A, Sheng JR, et al (2010) Nanodisc-incorporated hemagglutinin provides protective immunity against influenza virus infection. J Virol 84: 361–371. 19828606 10.1128/JVI.01355-09 PMC2798435

[32] Fischer NO, Infante E, Ishikawa T, Blanchette CD, Bourne N, et al (2010) Conjugation to nickel-chelating nanolipoprotein particles increases the potency and efficacy of subunit vaccines to prevent West Nile encephalitis. Bioconjug Chem 21: 1018–1022. 20509624 10.1021/bc100083d PMC2918428

[33] Weilhammer DR, Blanchette CD, Fischer NO, Alam S, Loots GG, et al (2013) The use of nanolipoprotein particles to enhance the immunostimulatory properties of innate immune agonists against lethal influenza challenge. Biomaterials 34: 10305–10318. 24075406 10.1016/j.biomaterials.2013.09.038 PMC7172747

[34] Fischer NO, Blanchette CD, Chromy BA, Kuhn EA, Segelke BW, et al (2009) Immobilization of His-tagged proteins on nickel-chelating nanolipoprotein particles. Bioconjug Chem 20: 460–465. 19239247 10.1021/bc8003155

[35] Rensen PC, de Vrueh RL, Kuiper J, Bijsterbosch MK, Biessen EA, et al (2001) Recombinant lipoproteins: lipoprotein-like lipid particles for drug targeting. Adv Drug Deliv Rev 47: 251–276. 11311995 10.1016/s0169-409x(01)00109-0

[36] Chromy BA, Arroyo E, Blanchette CD, Bench G, Benner H, et al (2007) Different apolipoproteins impact nanolipoprotein particle formation. J Am Chem Soc 129: 14348–14354. 17963384 10.1021/ja074753y

[37] Blanchette CD, Fischer NO, Corzett M, Bench G, Hoeprich PD (2010) Kinetic analysis of his-tagged protein binding to nickel-chelating nanolipoprotein particles. Bioconjug Chem 21: 1321–1330. 20586461 10.1021/bc100129s

[38] Blanchette CD, Law R, Benner WH, Pesavento JB, Cappuccio JA, et al (2008) Quantifying size distributions of nanolipoprotein particles with single-particle analysis and molecular dynamic simulations. J Lipid Res 49: 1420–1430. 18403317 10.1194/jlr.M700586-JLR200

[39] Rollett A, Reiter T, Nogueira P, Cardinale M, Loureiro A, et al (2012) Folic acid-functionalized human serum albumin nanocapsules for targeted drug delivery to chronically activated macrophages. Int J Pharm 427: 460–466. 22374516 10.1016/j.ijpharm.2012.02.028

[40] Gref R, Minamitake Y, Peracchia MT, Trubetskoy V, Torchilin V, et al (1994) Biodegradable long-circulating polymeric nanospheres. Science 263: 1600–1603. 8128245 10.1126/science.8128245

[41] Nguyen CA, Allemann E, Schwach G, Doelker E, Gurny R (2003) Cell interaction studies of PLA-MePEG nanoparticles. Int J Pharm 254: 69–72. 12615412 10.1016/s0378-5173(02)00685-3

[42] Lynch I, Cedervall T, Lundqvist M, Cabaleiro-Lago C, Linse S, et al (2007) The nanoparticle-protein complex as a biological entity; a complex fluids and surface science challenge for the 21st century. Adv Colloid Interface Sci 134–135: 167–174. 10.1016/j.cis.2007.04.021 17574200

[43] Salvati A, Pitek AS, Monopoli MP, Prapainop K, Bombelli FB, et al (2013) Transferrin-functionalized nanoparticles lose their targeting capabilities when a biomolecule corona adsorbs on the surface. Nat Nanotechnol 8: 137–143. 23334168 10.1038/nnano.2012.237

[44] Gao T, Blanchette CD, He W, Bourguet F, Ly S, et al (2011) Characterizing diffusion dynamics of a membrane protein associated with nanolipoproteins using fluorescence correlation spectroscopy. Protein Sci 20: 437–447. 21280134 10.1002/pro.577 PMC3048428

[45] Lattuada M, Hatton TA (2007) Functionalization of monodisperse magnetic nanoparticles. Langmuir 23: 2158–2168. 17279708 10.1021/la062092x

[46] Corbierre MK, Cameron NS, Lennox RB (2004) Polymer-stabilized gold nanoparticles with high grafting densities. Langmuir 20: 2867–2873. 15835165 10.1021/la0355702

[47] Jia H, Titmuss S (2009) Polymer-functionalized nanoparticles: from stealth viruses to biocompatible quantum dots. Nanomedicine (Lond) 4: 951–966. 19958231 10.2217/nnm.09.81

[48] Ackermann L, Potukuchi HK, Landsberg D, Vicente R (2008) Copper-catalyzed “click” reaction/direct arylation sequence: modular syntheses of 1,2,3-triazoles. Org Lett 10: 3081–3084. 18549230 10.1021/ol801078r

[49] Karver MR, Weissleder R, Hilderbrand SA (2012) Bioorthogonal reaction pairs enable simultaneous, selective, multi-target imaging. Angew Chem Int Ed Engl 51: 920–922. 22162316 10.1002/anie.201104389 PMC3304098

[50] Simon M, Zangemeister-Wittke U, Pluckthun A (2012) Facile double-functionalization of designed ankyrin repeat proteins using click and thiol chemistries. Bioconjug Chem 23: 279–286. 22188139 10.1021/bc200591x

[51] Zhang P, Liu S, Gao D, Hu D, Gong P, et al (2012) Click-Functionalized Compact Quantum Dots Protected by Multidentate-Imidazole Ligands: Conjugation-Ready Nanotags for Living-Virus Labeling and Imaging. Journal of the American Chemical Society 10.1021/ja302367s 22568447

[52] Trang P, Weidhaas JB, Slack FJ (2008) MicroRNAs as potential cancer therapeutics. Oncogene 27 Suppl 2: S52–57. 19956180 10.1038/onc.2009.353 PMC10033140

[53] Goodman CM, McCusker CD, Yilmaz T, Rotello VM (2004) Toxicity of gold nanoparticles functionalized with cationic and anionic side chains. Bioconjug Chem 15: 897–900. 15264879 10.1021/bc049951i

[54] Murphy CJ, Gole AM, Stone JW, Sisco PN, Alkilany AM, et al (2008) Gold nanoparticles in biology: beyond toxicity to cellular imaging. Acc Chem Res 41: 1721–1730. 18712884 10.1021/ar800035u

[55] Chen HT, Neerman MF, Parrish AR, Simanek EE (2004) Cytotoxicity, hemolysis, and acute in vivo toxicity of dendrimers based on melamine, candidate vehicles for drug delivery. Journal of the American Chemical Society 126: 10044–10048. 15303879 10.1021/ja048548j

[56] Fotakis G, Timbrell JA (2006) In vitro cytotoxicity assays: comparison of LDH, neutral red, MTT and protein assay in hepatoma cell lines following exposure to cadmium chloride. Toxicol Lett 160: 171–177. 16111842 10.1016/j.toxlet.2005.07.001

[57] Rubin EM, Ishida BY, Clift SM, Krauss RM (1991) Expression of human apolipoprotein A-I in transgenic mice results in reduced plasma levels of murine apolipoprotein A-I and the appearance of two new high density lipoprotein size subclasses. Proc Natl Acad Sci U S A 88: 434–438. 1703299 10.1073/pnas.88.2.434 PMC50825

[58] Ziv O, Avtalion RR, Margel S (2008) Immunogenicity of bioactive magnetic nanoparticles: natural and acquired antibodies. J Biomed Mater Res A 85: 1011–1021. 17924557 10.1002/jbm.a.31518

[59] Ochsenbein AF, Zinkernagel RM (2000) Natural antibodies and complement link innate and acquired immunity. Immunol Today 21: 624–630. 11114423 10.1016/s0167-5699(00)01754-0

[60] Irngartinger M, Camuglia V, Damm M, Goede J, Frijlink HW (2004) Pulmonary delivery of therapeutic peptides via dry powder inhalation: effects of micronisation and manufacturing. Eur J Pharm Biopharm 58: 7–14. 15207532 10.1016/j.ejpb.2004.03.016

[61] Courrier HM, Butz N, Vandamme TF (2002) Pulmonary drug delivery systems: recent developments and prospects. Crit Rev Ther Drug Carrier Syst 19: 425–498. 12661699 10.1615/critrevtherdrugcarriersyst.v19.i45.40

[62] Azarmi S, Roa WH, Lobenberg R (2008) Targeted delivery of nanoparticles for the treatment of lung diseases. Adv Drug Deliv Rev 60: 863–875. 18308418 10.1016/j.addr.2007.11.006

[63] Niven RW (1995) Delivery of biotherapeutics by inhalation aerosol. Crit Rev Ther Drug Carrier Syst 12: 151–231. 9501969 10.1615/critrevtherdrugcarriersyst.v12.i2-3.20

